# Activity and Mechanism of Action of the Bioceramic Silicon Nitride as an Environmentally Friendly Alternative for the Control of the Grapevine Downy Mildew Pathogen *Plasmopara viticola*

**DOI:** 10.3389/fmicb.2020.610211

**Published:** 2020-12-14

**Authors:** Giuseppe Pezzotti, Yuki Fujita, Francesco Boschetto, Wenliang Zhu, Elia Marin, Elodie Vandelle, Bryan J. McEntire, Sonny B. Bal, Marco Giarola, Koichi Makimura, Annalisa Polverari

**Affiliations:** ^1^Ceramic Physics Laboratory, Kyoto Institute of Technology, Kyoto, Japan; ^2^Department of Immunology, Graduate School of Medical Science, Kyoto Prefectural University of Medicine, Kyoto, Japan; ^3^Department of Orthopedic Surgery, Tokyo Medical University, Tokyo, Japan; ^4^The Center for Advanced Medical Engineering and Informatics, Osaka University, Osaka, Japan; ^5^Department of Dental Medicine, Graduate School of Medical Science, Kyoto Prefectural University of Medicine, Kyoto, Japan; ^6^Laboratory of Phytopathology, Department of Biotechnology, University of Verona, Verona, Italy; ^7^SINTX Technologies Corporation, Salt Lake City, UT, United States; ^8^Raman Laboratory, Centro Piattaforme Tecnologiche, University of Verona, Verona, Italy; ^9^Medical Mycology, Graduate School of Medicine, Teikyo University, Tokyo, Japan

**Keywords:** silicon nitride, *Plasmopara viticola*, bioactive, environmentally friendly, grapevine

## Abstract

Downy mildew of grapevine, caused by *Plasmopara viticola* (Berk. and Curt.) Berl. and de Toni, is one of the most devastating diseases of grapevine, severely affecting grape and wine production and quality worldwide. Infections are usually controlled by the intensive application of synthetic fungicides or by copper-based products in organic farming, rising problems for soil contamination and adverse impacts on environment and human health. While strict regulations attempt to minimize their harmful consequences, the situation calls for the development of alternative fungicidal strategies. This study presents the unprecedented case of a bioceramic, silicon nitride, with antimicrobial properties against *P. viticola*, but without adverse effects on human cells and environment, opening the way to the possible extension of silicon nitride applications in agriculture. Raman spectroscopic assessments of treated sporangia in conjunction with microscopic observations mechanistically showed that the nitrogen-chemistry of the bioceramic surface affects pathogen’s biochemical components and cell viability, thus presenting a high potential for host protection from *P. viticola* infections.

## Introduction

The interest of consumers toward sustainability of food production is an important issue and sustainable practices in plant disease control are often considered as quality indicators (Schäufele and Hamm, 2017). In particular, grapevine is one of the crops with the strongest environmental impact: nearly two-thirds of all applied synthetic fungicides in the European Union are used to control grapevine diseases (Muthmann and Nardin, 2007), especially powdery and downy mildew, caused by the ascomycete *Erysiphe necator* and the oomycete *Plasmopara viticola*, respectively (Armijo et al., 2016).

Native to North America and accidentally introduced into Europe at the end of the last century, the oomycete *Plasmopara viticola* (Berk. and Curt.) Berl. and de Toni can only be controlled in conventional viticulture through multiple annual applications of synthetic chemical compounds, or by copper-based products in organic farming (Gessler et al., 2011). Applied research programs and legislative measures have largely improved the situation in the last decades. Many phytosanitary products with unacceptable eco-toxicological profiles have been removed from the market; even copper, which is still allowed in organic farming, was recently included in the list of candidates for substitution (European Commission Implementing Regulation 2018/84) and its use is being progressively restricted (Reganold and Wachter, 2016). Besides breeding for genetic resistant varieties (Töpfer and Eibach, 2016), some low-impact alternatives to currently used fungicides have been proposed, ranging from natural antimicrobial extracts to antagonistic microorganism for biocontrol and resistance inducers that boost the natural plant defense capacity (Perazzolli et al., 2008; Dagostin et al., 2011), but none of these strategies is still fully capable to protect plants from this serious disease.

*P. viticola* attacks all parts of the plant including leaves and young fruit (Gessler et al., 2011). Assisted by temperate and humid weather, asexual sporangia release zoospores which eventually attach to and encyst on stomata to form a penetrating germ tube that gives rise to an infection vesicle into the substomatal cavity. A primary hypha emerges and quickly develops branches whose haustoria penetrate plant cells to draw nutrients. After several days of infective incubation, the sporangiophores emerge to release new sporangia (Burruano, 2000; Kiefer et al., 2002). A broad spectrum of phytosanitary treatments are applied during the growing season, depending on the stage of the disease, the grapevine variety, and environmental conditions (Caffi et al., 2010). In most geographic areas, management of downy mildew requires several applications, starting at the beginning of vegetative development (Gessler et al., 2011). The frequent use of fungicides, their high cost, their long-term harm to the environment and the selection of resistant strains in the pathogen population, call for the development of more effective, long-lasting, and eco-friendly strategies, in an integrated pest management system that includes all possible control measures, restricting the use of conventional pesticides to conditions where they are actually needed.

In the attempt to find new alternatives that could be developed into effective, environmentally safe agrochemicals, we have investigated the ability of silicon nitride (Si_3_N_4_), to knockdown *P. viticola* starting early in its infection cycle. The choice of this ceramic was based on its unique surface chemistry within an aqueous environment (Pezzotti, 2019) and its antibacterial (Pezzotti et al., 2018), antiviral (Pezzotti et al., 2020) and antifungal properties (Pezzotti, under review) while still being harmless to humans and widely used as a new therapeutic bioceramics in osteoarthropathy (Pezzotti et al., 2017; Dai et al., 2019; Calvert et al., 2020). Here we explore the possibility to consider Si_3_N_4_) as a possible alternative for grapevine protection against *P. viticola*. Moreover, we investigated its mechanism of action by microscopy and *in situ* Raman spectroscopy, a non-invasive method that can be applied to living pathogens without markers, in time-lapse experiments, to monitor sporangial chemical structures and their evolution during chemical interactions with Si_3_N_4_ (Pezzotti, 2019). This study thus lays the foundation for a field experiment using Si_3_N_4_ as an eco-friendly antifungal alternative to existing agrochemicals.

## Materials and Methods

### Sample Preparation

*P. viticola* isolate harvested in 2019 was axenically grown as described by Polesani et al. (2010). To evaluate the possible phytotoxicity of Si_3_N_4_, treatments were performed using two different grape varieties, Cabernet Sauvignon and Cannonau. Cabernet Sauvignon leaves were taken from 3 year-old plants, while Cannonau leaves were obtained from young seedlings grown in a greenhouse under controlled conditions (16 h light/8 h dark, temperature range 18–28°C).

Si_3_N_4_ powder (SINTX Technologies, Salt Lake City, UT, United States) with an average particle size of ∼2 μm was used. It was obtained by grinding sintered ß-Si_3_N_4_ powder having a nominal composition of 90 wt.% α-Si_3_N_4_, 6 wt.% yttrium oxide (Y_2_O_3_), and 4 wt.% aluminum oxide (Al_2_O_3_). The constituents were sintered at ∼1,700°C for > 3 h and hot-isostatically pressed at about 1,600°C for 2 h. After preparation, it was heat sterilized at 180°C for 2 h before suspension in sterile distilled water.

For evaluation of preventive efficacy, three lots of five disks (∼3 cm in diameter) were cut from sterilized leaves for each grape variety. One lot was treated by full immersion in a 1.5 vol.% aqueous suspension of Si_3_N_4_ for 1 min and inoculated with 40 μL of germinated sporangia suspension (3 × 10^4^/mL) 24 h later (pre-treated samples). A second lot was exposed to sporangia combined with the 1.5 vol.% Si_3_N_4_ suspension. In this case, the Si_3_N_4_ granules remained in direct contact with the sporangia during germination (co-treated samples). The third lot was inoculated with *P. viticola* and served as an infection control group. All disks were incubated in a growth chamber at 21–24°C with a day/night photoperiod of 16 and 8 h, respectively, and monitored for 6 days until sporulation appeared on the controls.

### Microscopy Observation

Sporangia suspended in water or the 1.5 vol.% Si_3_N_4_ suspension (3 × 10^4^ sporangia/ml) were observed under an epifluorescence microscope (Leica DM/RB, excitation filter BP 340–380 nm; dichroic mirror 400 nm; suppression filter LP > 430 nm) or stained with 0.05 μg/μL of Fluorescein diacetate (FDA) and observed using a fluorescence microscope (Leica DFC350FX) to check sporangia viability during a time course of 3 h. Observations were made in a cell counting B*ü*rker chamber to calculate the percentage of viable sporangia in comparison to water-treated controls.

### pH Measurements

The pH of sterile double distilled water was measured with a pH-meter (PP-20; Sartorius, New York, United States) after the addition of 1.5 vol.% Si_3_N_4_ powder. Measurements were made while stirring at room temperature as a function of time for up to 800 s at intervals of 10 s until final pH stabilization. To check whether the pH trend was reproducible, the tested powder sample was separated by centrifugation (13 × 10^3^ rpm for 3 min) and dried at 180°C in air for 2 h. After cooling to room temperature, the powder was re-suspended at the same concentration in water (i.e., 1.5 vol.%) for additional pH measurements. The procedure was repeated with the same powder for three subsequent cycles.

### *In situ* Raman Spectroscopy

In situ Raman spectra were collected on sporangia samples suspended in water with and without Si_3_N_4_ powder. Raman spectra were obtained using a dedicated instrument (DXR2, Thermo Fisher Scientific, Inc., Madison, WI) operating in microprobe mode with a 50x optical lens. The spectroscope was equipped with a holographic notch filter, which concurrently allowed high-efficiency and high-resolution spectral acquisitions. Excitation was made with a 785 nm laser source at a power max of 15 mW. The Raman scattered light was monitored using a single monochromator connected with an air-cooled charge-coupled device (CCD) detector. The acquisition time of one spectrum was typically 60 s. The spectra for different sporangia samples were averaged over ∼10 different collection locations. Raman spectra were deconvoluted into Gauss-Lorentz cross-product sub-band components using commercially available software (LabSpec 4.02, Horiba/Jobin-Yvon, Kyoto, Japan). Spectral band assignments were made according to published literature.

## Results

### *pH* Analyses of Si_3_N_4_ Powder in Aqueous Suspensions

**FIGURE 1:**
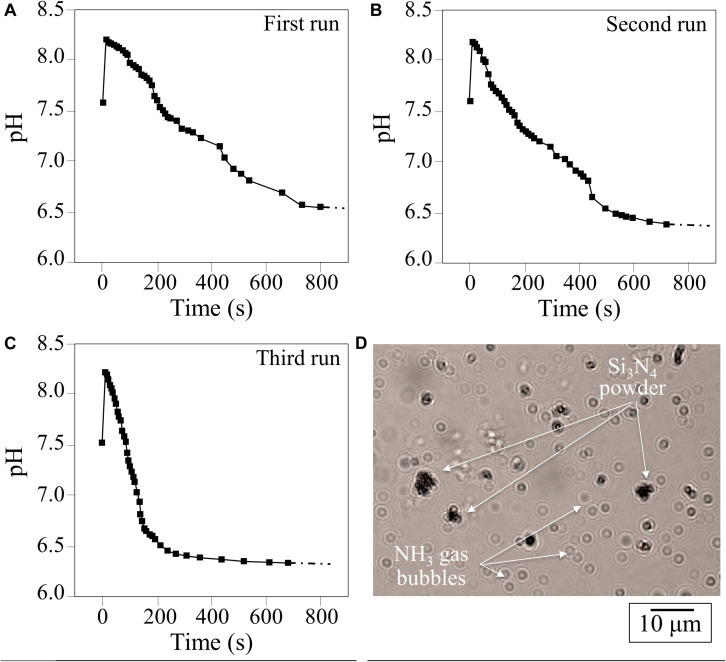
**(A–C)**, results of three successive runs of pH measurements as a function of time in a water suspension of 1.5 vol.% Si_3_N_4_ micrometer-sized particles; **(D)** observation of NH_3_ gas bubbles produced in water shortly after dispersion of Si_3_N_4_ powder.

The change in pH as a function of time for 1.5 vol.% Si_3_N_4_ water suspension is shown in Figure 1. This pH experiment was conceived to simulate the effect of periodic rain in grapevine fields after having been sprayed with a dose of Si_3_N_4_ powder. Three successive repetitive trials involving suspension, measurement, and drying are given in A, B, and C, respectively. Independent of run sequence, the plots showed a sudden (within seconds) increase in pH from an initial neutral value (pH∼7.5) to a maximum (pH∼8.3). The curves for the first and second runs were very similar, while the third run showed a steeper reduction over time, although the plateau (pH∼6.3–6.7) was similar for all trials. This phenomenon, which was characterized in a previous study using pH microscopy and a colorimetric ammonia assay (Pezzotti, 2019), is associated with the cleavage of the Si-N bond at the Si_3_N_4_‘s surface and the reaction of eluted nitrogen with hydrogen to form ammonia (NH_3_) or ammonium (NH_4_^+^). In an open system, a gradual drop in pH to 6.3***–***6.7 takes place in about 5 min. Since the fraction of NH_3_ in solution is inversely dependent on pH, the time-dependent data suggest that an increasing fraction of highly volatile NH_3_ leaves the aqueous system. This was confirmed by direct observation of gas bubbles produced shortly after the dispersion of Si_3_N_4_ powder (Figure 1D). At pH∼8.3, the fraction of NH_3_ was computed to be 7***–***10%, while in the acidic solution it was nearly zero (Pezzotti, 2019). These results show that the same Si_3_N_4_ powder can provide prolonged pH buffering during sequential rain events, assuming that a fraction of the Si_3_N_4_ powder could remain attached to the leaf rugosity or entrapped into the leaf stomatal cavities.

### *In situ* Microscopic Monitoring of Sporangia*/*Si_3_N_4_ Granule Interaction

**FIGURE 2:**
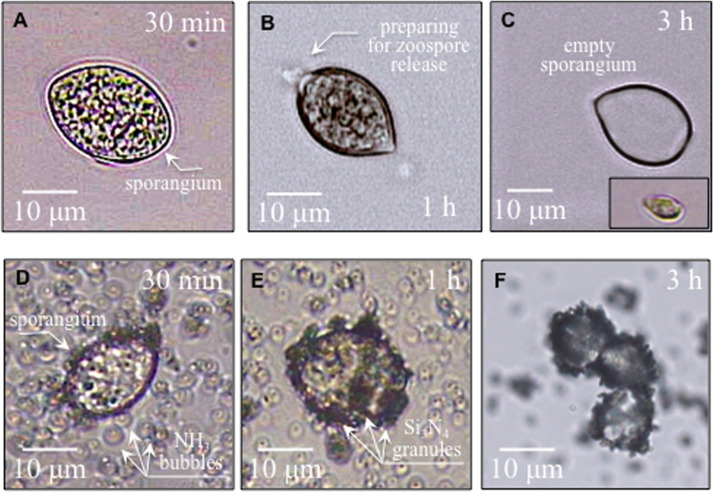
In **(A,B)**, micrographs of sporangia in pure sterile distilled water progressively preparing for zoospore release; in **(C)**, and empty sporangium after germination and a released zoospore in the lower right inset. In **(D–F)**, sporangia interacting with Si_3_N_4_ granules in water suspension (1.5 vol.%) gradually becoming covered with Si_3_N_4_ granules without releasing zoospores.

Figures 2A–C show time-lapse micrographs of sporangia in water environment, in which sporangia gradually prepared for germination and finally quickly discharged swimming zoospores (i.e., about 3 h later), as detailed in Figure 2C (cf. also inset micrograph). Similar time-lapse micrographs are given in Figures 2D–F for sporangia interacting with granules of Si_3_N_4_ powder in water suspension. The Si_3_N_4_ appeared to be electrostatically attracted to the external wall of the sporangium. Contact occurred almost immediately upon the introduction of Si_3_N_4_ into the sporangia suspension (10^5^ ml**^–^**^1^) and gradually increased until the whole sporangium surface was fully covered by Si_3_N_4_ granules, with a possibly almost complete block of germination, as mobile zoospores were never detected in treated samples.

Collectively, these micrographs revealed that an aggressive environment developed in the vicinity of the sporangia. The presence of ammonia gas bubbles coupled with increased pH, as shown in Figure 1D, is related to the production of gaseous ammonia, a volatile molecule that could easily penetrate the sporangia’s cell wall. Similarly, fungal cells are permeable to ammonia, which enters the cells by the free diffusion of the undissociated molecule (MacMillan, 1956).

**FIGURE 3:**
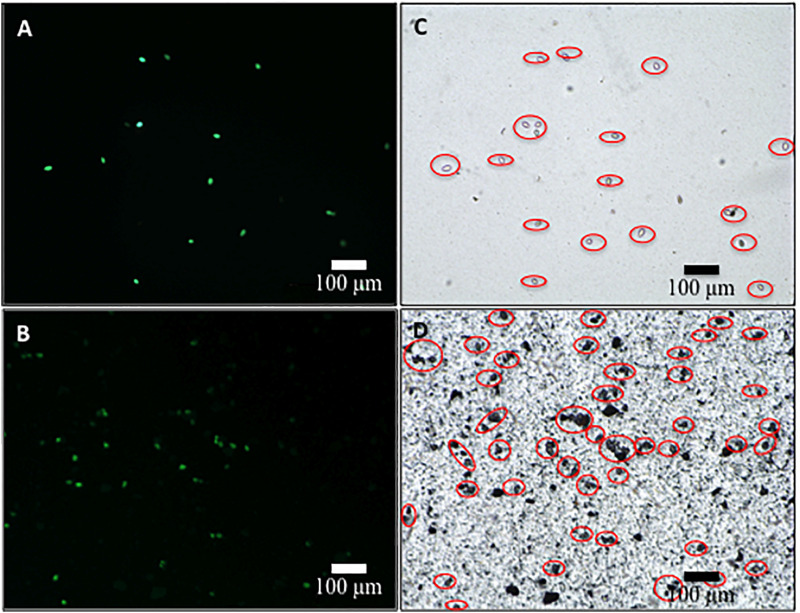
Fluorescence images of sporangia suspended in water (3.0 × 10^4^ mL^– 1^) for 3 h without **(A)** and with **(B)** 1.5 vol.% Si_3_N_4_ granules (stain was made with fluorescein diacetate in concentration 0.05 μg/μL); in **(C,D)**, light microscopy images of the same optical fields showing the total number of sporangia in the samples.

**FIGURE 4:**
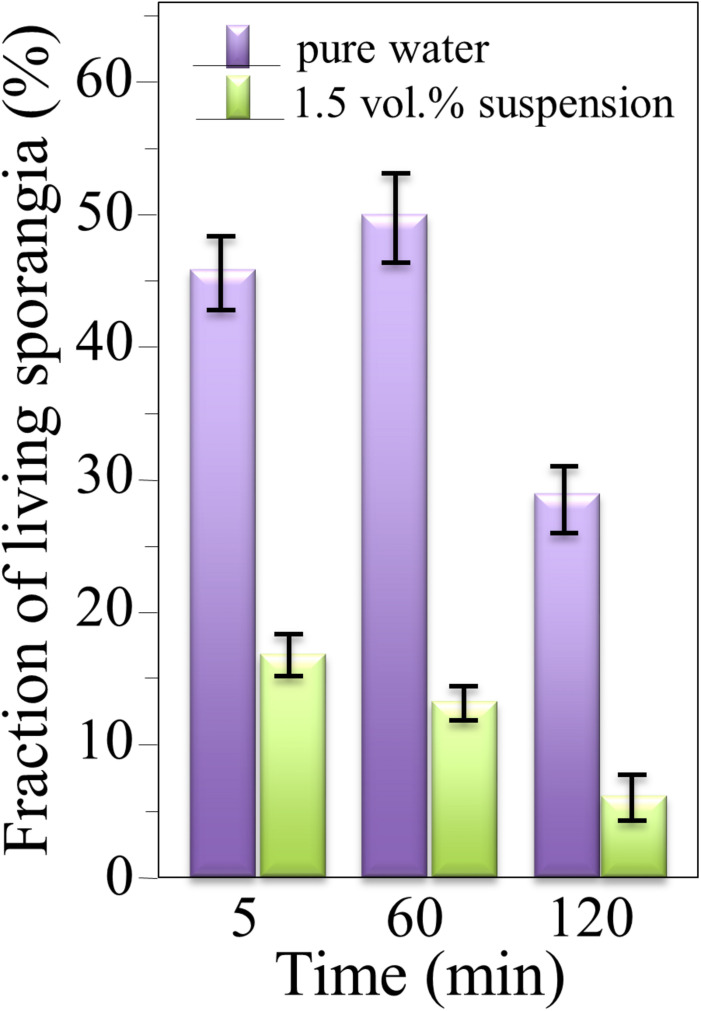
Fractions of living sporangia after incubation in pure water or in a suspension of 1.5 vol.% Si_3_N_4_ granules in water up to 3 h, calculated upon comparing fluorescence and light microscopy images; > 100 sporangia were counted for each sample (bars show standard deviations computed on 3 independent experiments).

The survival of sporangia in contact with Si_3_N_4_ granules was monitored by optical and fluorescence microscopies in order to quantify the antimicrobial effect of the ceramic. Figures 3A,B show fluorescence images of a suspension of living sporangia (10^5^ ml^–1^), suspended in water for 3 h without and with 1.5 vol.% Si_3_N_4_ granules, respectively, and then stained with fluorescein diacetate. The concurrent quantifications by direct counting of the number of living sporangia on fluorescence images and of the total number of sporangia by light microscopy (in Figures 3C,D) resulted in the fractional plot shown in Figure 4. A clear reduction of sporangia viability was observed for the suspension containing the Si_3_N_4_ granules when compared to pure water.

### Monitoring Preventive Si_3_N_4_ Efficacy Against *P. viticola*

**FIGURE 5:**
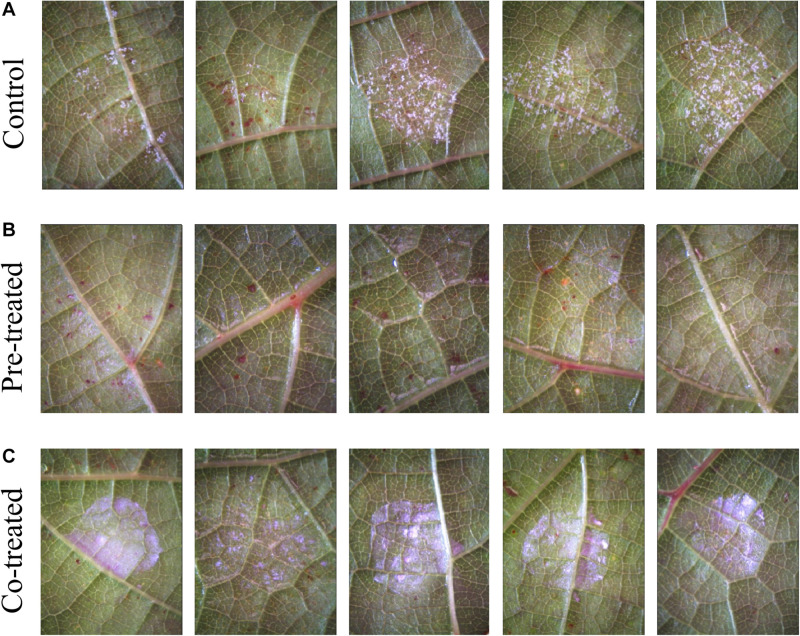
Efficacy of 1.5 vol.% Si_3_N_4_ powder suspension in preventing *P. viticola* infection on grapevine leaves cv. Cabernet Sauvignon: **(A)** Non-treated samples showing signs of pathogen sporulation after artificial infection on 5 different leaves; **(B)** complete protection from pathogen infection on leaves pre-treated with 1.5 vol.% Si_3_N_4_ 24 h before inoculation; and **(C)** complete protection in co-treated samples. Whitish-violet haloes are due to residual granules of Si_3_N_4_.

**FIGURE 6:**
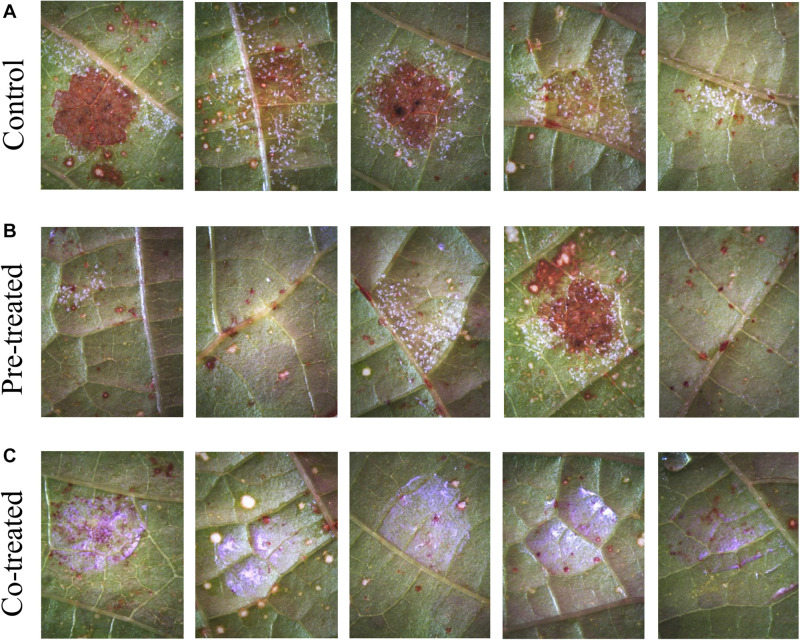
Efficacy of 1.5 vol.% Si_3_N_4_ powder suspension in preventing *P. viticola* infection on grapevine leaves cv. Cannonau species: **(A)** Non-treated samples showing signs of pathogen sporulation and leaf necrosis after artificial infection on 5 different leaves; **(B)** strong protection from pathogen infection on 3 over 5 leaves pre-treated with 1.5 vol.% Si_3_N_4_ 24 h before inoculation; and **(C)** complete protection in co-treated samples. Whitish-violet haloes are due to residual granules of Si_3_N_4_.

The experimental results on leaf-disks from the two grapevine cultivars (cv.), Cabernet Sauvignon and Cannonau, are shown in Figures 5, 6, respectively. Visual inspection of treated uninfected leaves at the stereomicroscope did not reveal signs of phytotoxicity along the 5 days of the experiment. In both cases, five leaf-disk samples for each of two experimental runs were tested (*n* = 10), with both experimental runs showing similar results. Control samples were concurrently examined (panels A in both Figures 5, 6). All control leaf-disks showed infections. Pathogen sporulation on Cabernet Sauvignon leaf-disks occurred 5 days after inoculation (Figure 5A). For cv. Cannonau, the infection on the control leaf-disks was more severe (Figure 6A), in most cases resulting in necrosis of the infected spots. This is often observed in highly susceptible cultivars or young tender leaf tissues (Steimetz et al., 2012). Complete protection from infection was observed for samples of cv. Cabernet Sauvignon that were either pre-treated or co-treated with Si_3_N_4_ (Figures 5B,C, respectively). For the cv. Cannonau species, strong or total protection was only observed in 3 out of 5 leaf-disks in pre-treated samples (Figure 6B), whereas no signs of sporulation were seen in co-treated disks (Figure 6C).

### *In situ* Raman Spectroscopic Monitoring of Sporangia

**FIGURE 7:**
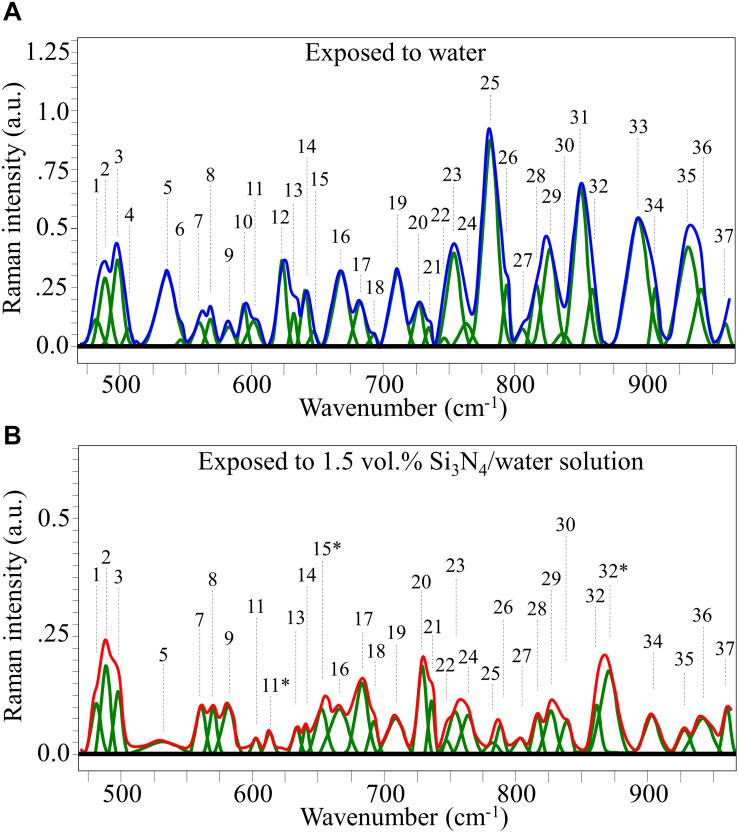
Average Raman spectrum of *P. viticola* after immersion for 10 min at room temperature in pure water **(A)** and in a water suspension containing 1.5 vol.% Si_3_N_4_ powder **(B)**; spectra were normalized to the glucans’ signal at 424 cm^– 1^ (not shown), which showed similar intensity in the compared spectra. The bands labeled with an asterisk in **(B)** represent signals that newly appeared after exposure of the pathogen to the Si3N4 powder in water suspension.

Raman spectra of *P. viticola*, collected after short-term room-temperature immersion in pure water and the suspension containing 1.5 vol.% Si_3_N_4_ powder are shown in Figures 7A,B, respectively. The spectra were normalized for the scatter intensity observed at 424 cm^–1^ (not shown). This signal, which represents skeletal vibrations, is a non-specific marker common to all glucans (Corbett et al., 1991). It did not show an appreciable difference in intensity for aqueous exposure with and without the Si_3_N_4_ powder. The spectra were then deconvoluted into Voigtian sub-bands and compared. The clear differences between the two spectra are due to changes in the structure of the oomycete. The deconvoluted Raman bands for each spectrum are shown in Figure 7, and a list of possible assignments and references are listed in Supplementary Table S1.

#### Spectra of Sporangia Exposed to Pure Water

Oomycetes have recently been re-classified in *Stramenopiles* according to an updated classification (Adl et al., 2019). Main structural characteristics include the presence of cellulose in the wall, mycolaminarine instead of glycogen as a carbon-based energy source, and a conspicuous lack of chitin. Recent analyses of carbohydrate content in the oomycete *Phytophthora parasitica*, closely related to *P. viticola*, revealed that the cell walls were completely devoid of chitin and consisted by ∼85% of β-glucans, about 40% of which was represented by cellulose. 1,3 β-glucans with low polymerization level, and 1,3,6 β-glucans were also present, together with lower fractions of glucuronic acid and mannan (Melida et al., 2013). Such detailed information is not available for *P. viticola*, but previous evidences indicate that this pathogen might slightly differ from other best-known organisms in the *Peronosporales*. Indeed, *P. viticola* can express at least two different chitin synthases, and chitin was detected on the surface of sporangia, sporangiophores, and hyphal cell walls during *in planta* growth (Werner et al., 2002; Martinez-Prada and Kortekamp, 2015).

Non-germinated sporangia, as in the case of the present Raman assessments, are typical syncytial cells with multiple nuclei and mitochondria nested in a dense cytoplasm, with small early dense-body vesicles, possibly composed of lipids, which have been proposed as energy sources for the subsequent steps of cellularization and hatching (Troester et al., 2017). Negrel et al. (2018) recently searched for specific metabolic markers of *P. viticola* presence in infected grapevine leaves and identified three types of atypical lipids—ceramides, and derivatives of arachidonic and eicosapentaenoic acids, produced by the pathogen since the very early stage of its development.

According to the above notions, we searched for specific structural features in the Raman spectrum of Figure 7A (*cf*. also Supplementary Table S1 and references cited therein). As specified above, similar to other oomycete structures, the cell walls of *P. viticola* mainly consist of polysaccharides, including branched polymeric glucose-containing β-glucans, and polymeric mannose covalently associated with glyco/manno-proteins. Proteins and lipids only represent minor fractions of the total compared to polysaccharides. Accordingly, carbohydrate vibrational modes are expected to dominate the Raman spectrum. Cumulative signals from backbone glucose rings were found in Band 1 at 482 cm^–1^ (De Gussem et al., 2005). Bands 14, 15, and 19 at 643, 649, and 710 cm^–1^, respectively, relates to = C-H bending, while Band 33 at 893 cm^–1^ originates from C-H ring stretching (De Gussem et al., 2005; Machovic et al., 2017). These bands could all be related to chitin, although chitin is actually expected to be a minor component among the carbohydrates of the studied oomycete sample. A more probable assignment for Bands 19 and 33 is cellulose, while Bands 14 and 15 could both be also assigned to β-D-glucose in linear polymer cellulose (De Gussem et al., 2005). An additional fingerprint signal from cellulose could be found at 583 cm^–1^ (Band 9; C-C-O bending and C-O torsional vibrations) (De Gussem et al., 2005). As expected from the structure of the walls, a marked signal was found at 893 cm^–1^ (equatorial C-H bending vibrations), which served as a marker for β-glucans (Cael et al., 1974; Noothalapati et al., 2016). The absence of Raman signals at 550 cm^–1^ (C-O-C bending of glycosidic linkage), which is a fingerprint vibration for α-glucans, is consistent with the notion that this polysaccharide isomer is not present in oomycete cell walls. For this reason, any contribution from the α-glucans to the Raman signal at 942 cm^–1^ (anti-symmetric ring vibrations) was excluded from the spectrum of Figure 7A. Similar reasoning was applied to ring vibrations from the dextran structure, which should occur at 922 cm^–1^, and its glycosidic signals at 550 cm^–1^. Neither of these signals was detected in the spectrum of *P. viticola* exposed to water. These observations fit with the absence of this complex glucan in the studied oomycete structure. In the narrow spectral region between 490 and 560 cm^–1^ in Figure 7A, Bands 2 and 3 (at 500 and 558 cm^–1^, respectively) are mainly signals from C-C backbone stretching in polysaccharides and D(+)-mannose, respectively (Williams and Edwards, 1994), while Bands 4 (very weak), 5, and 7 (at 510, 535, and 558 cm^–1^, respectively) were assigned to cellulose, trehalose (ring deformation), and β-D-glucose, respectively (Supplementary Table S1; Cael et al., 1974; She et al., 1974). The disaccharide trehalose is the main contributor of Band 11 at 603 cm^–1^, and it also contributes Bands 6 and 30 (at 544 and 837 cm^–1^, respectively) (De Gussem et al., 2005). Trehalose contributions to Bands 31 and 34 (at 846 and 906, respectively) are presumably of lower weight as compared to other carbohydrate structures (Supplementary Table S1). More specifically, the intensity of Band 31 is also contributed by the vibrations related to glucose and glucans, but it also contains several medium/strong signals from triglycerides (Supplementary Table S1). Trehalose is an important molecule in the metabolism of many species of microorganisms because it is an energy source and a protective molecule against environmental stress. For example, *Candida albicans* promotes the synthesis of non-reducing trehalose disaccharide and accumulates it in response to heat or oxidative stress (Lu et al., 2011). In grapevine, *P. viticola* is known to induce irreversible stomatal opening, which in turn favors host infection by zoospores, and this deregulation is associated with trehalose accumulation (Gamm et al., 2015), with exogenously applied trehalose antagonizing stomatal closure. Therefore, the presence of elevated levels of trehalose in sporangia may represent a signal facilitating infection of grapevine leaves.

Signals related to nucleic acid were found from both phosphodiester and purine bonds. C’5-O-P-O-C’3 phosphodiester bond symmetric stretching in DNA (Band 25 at 782 cm^–1^) was the strongest signal detected in the low-frequency spectrum of *P. viticola* exposed to pure water (Figure 7A; Notingher et al., 2004). Unlike this individual signal, the corresponding antisymmetric stretching Band 29 at 827 cm^–1^ might have overlapped with several signals from sterols, typical molecules in cell membranes (Supplementary Table S1 and references therein). Vibrational bands from purines were also observed, which were related to adenine (Bands 5, 12, 21, and 36 at 535, 623, 731, and 942 cm^–1^, respectively) (Lopes et al., 2013), cytosine (Bands 6, 7, 10, 11, 19, and 26 at 544, 558, 594, 603, 710, and 795 cm^–1^, respectively) (Mathlouthi and Seuvre, 1986a), guanine (Bands 8, 14, 17, and 31 at 570, 643, 681, and 846 cm^–1^, respectively) (Mathlouthi and Seuvre, 1986b), thymine (Bands 12, 22, and 23 at 623, 746, and 753 cm^–1^, respectively) (Mathlouthi and Seuvre, 1984; Gamm et al., 2015), and uracil (Band 27 at 807 cm^–1^) (Madzharova et al., 2016). Band 31 at 846 cm^–1^, which is the second strongest detected signal in the studied frequency range (Figure 7) is predominantly contributed by C4-N9-C8 + N1-C2-N3 and N2-C2-N3 in-plane deformation of guanine rings (Lopes et al., 2012).

Looking for peculiar signals from lipids usually present in cell membranes, a strong emission from phosphatidylserine in the studied spectral area was expected at about 734 cm^–1^(Krafft et al., 2005). Band 21 was indeed observed at ∼731 cm^–1^ in the spectrum of sporangia exposed to pure water (Figure 7A). However, possible contributions to this band could also come from breathing of the imidazole ring in DNA adenine and from trehalose as well. Main bands from phosphatidylcholine in the low-frequency spectrum were expected at around 719 and 875 cm^–1^ (Krafft et al., 2005). However, in Figure 7A, neither of these signals could be observed in the sporangia exposed to pure water. The main low-frequency bands at 519 and 868 cm^–1^, which were due to phosphatidylinositol (Krafft et al., 2005), were also missing in the spectrum of water-exposed sporangia. Conversely, clear signals possibly from sterols (Krafft et al., 2005) and ceramides (Mishra and Tandon, 2012) at 558 (Band 7) and 681 cm^–1^ (Band 17), respectively, were detected. Unfortunately, also these signals strongly overlapped with signals from DNA purines.

Sterols are characterized by complex Raman spectra, which include clear low-frequency signals (Supplementary Table S1). However, an accurate screening revealed that none of these low-frequency signals was free of overlapping signals from other membrane molecules. Sterols are essential components in modulating fluidity, permeability, and integrity of the cell membranes. In contrast to true fungi, *Peronosporales* are unable to synthesize sterols, although they need them for both sexual and asexual reproduction (Elliot, 1983). In *Phytophthora*, fitosteroles from the plant host are taken up and used without any further modification (Dahlin et al., 2017).

Regarding other lipid compounds, arachidonic acid is a well-known elicitor released by oomycetes in planta (Ricker and Bostock, 1992) and recent findings indicate that ceramides and derivatives of arachidonic and eicosapentaenoic acid in *P. viticola* are produced during the very early stages of the infection process (Negrel et al., 2018), suggesting to assign Bands 32 (at 861 cm^–1^) and 35 (at 931 cm^–1^) to C-O vibrations in alpha-linolenic acid and C-H bending in arachidonic acid (Czamara et al., 2015), although this latter band unfortunately overlaps with bands from glucose and histidine (as described later). Band 20 at 715 cm^–1^ is considered to be the strongest signal of (C-N stretching) (De Gussem et al., 2005). Additional bands from lecithin appear at 764 and 827 cm^–1^ (Bands 24 and 29, respectively). These signals are attributed to O-P-O symmetric and antisymmetric stretching, respectively. An attempt to give a complete labeling of the complex spectrum shown in Figure 7A is given in Supplementary Table S1.

#### Spectra of Sporangia Exposed to 1.5 vol.% Si_3_N_4_ Water Suspension

Changes in the cellular structure *P. viticola* sporangia induced by the presence of Si_3_N_4_ in aqueous suspension are shown by the spectral variations between Figures 7A,B. As a general trend, all Raman Bands for the sporangia exposed to the Si_3_N_4_ suspension showed relatively lower intensities when compared to the corresponding bands of sporangia in pure water. The main variations noticed in the normalized spectra were as follows:

(i) Several bands of high or medium intensity disappeared or occurred only with significantly reduced intensity in the spectrum of sporangia in the Si_3_N_4_ suspension. They included: Bands 5 and 12 (at 535 and 623 cm^–1^, respectively) from adenine; Band 10 (at 594 cm^–1^) from cytosine; Band 25 (at 782 cm^–1^) from C’5-O-P-O-C’3 phosphodiester symmetric stretching in DNA; Band 31 (at 846 cm^–1^) from guanine; and, Band 33 (at 893 cm^–1^) from cellulose (possibly also contributed by chitin).
(ii) Three new bands appeared in the sporangia spectrum exposed to the Si_3_N_4_ suspension. They were: Band 11^∗^ (at 613 cm^–1^), Band 15^∗^ (at 654 cm^–1^), and Band 32^∗^ (at 872 cm^–1^). The origin of these Raman signals could be due to chemical modifications of pre-existing molecules or from new chemical species produced by the sporangia in response to environmental stress (as discussed later).
(iii) Additional spectral variations in the presence of Si_3_N_4_ regarded Band 2 from C-C backbone stretching in polysaccharides and Band 3 from D(+)-mannose (at 490 and 500 cm^–1^, respectively). These signals underwent an intensity-trend inversion, the former becoming more intense than the latter. In addition, Band 9 from cellulose (at 583 cm^–1^) also showed a relatively higher intensity. A similar trend was observed for Band 17 (at 681 cm^–1^), which was assigned to O = CN and CCO bending in ceramides (Mishra and Tandon, 2012), but also had contributions from the guanine ring. Band 23 (at 753 cm^–1^) representative of thymine, Band 35 from histidine (at 931 cm^–1^), and Band 36 from adenine (at 942 cm^–1^) experienced significant decreases in intensity.

The reasons for the bold spectral differences between sporangia exposed to pure water and the aqueous Si_3_N_4_-powder suspension will be discussed in the next section based to the assumption that such differences were the result of chemical reactions occurring between sporangia and the Si_3_N_4_ granules.

## Discussion

### The Chemical Interaction Between *P. viticola* and Si_3_N_4_

As described in two previously published studies (Pezzotti, 2018, 2019), the surface of Si_3_N_4_ undergoes homolytic dissociation of Si-N covalent bonds in an aqueous environment. The release of nitrogen and silicon is the chemical origin of Si_3_N_4_’s biological effectiveness. A hydrolysis process initiates the protonation of amino groups at the Si_3_N_4_ surface. The electrophilicity at the adjacent silicon sites increases, which in turn leads to the propensity of these sites to undergo nucleophilic attack by water. As water interacts with silicon sites, metastable pentacoordinated complexes first form, but then promptly decay with the liberation of ammonia/ammonium ions at a ratio that depends on the environmental pH (Laarz et al., 2000). The pH-dependent elution of ammonia (NH_3_) or ammonium (NH_4_^+^) takes place together with the formation of silicon dioxide (SiO_2_) on the solid surface. Hydrolysis of this latter species produces orthosilicic acid, Si(OH)_4_. The basic chemical reactions of Si_3_N_4_ in water are as follows:

(1) SiN3+46HO2→3SiO+24NH3

(2) H++NH→3NH+4

(3) SiO+22HO2→Si(OH)4

At room temperature, the fraction of NH_3_ varies with pH according to a sigmoidal dependence (Sawyer and McCarty, 1978; Parker et al., 1995).

(4) [NH]3[H]+/[NH]4+=5.7×100-1

**FIGURE 8:**
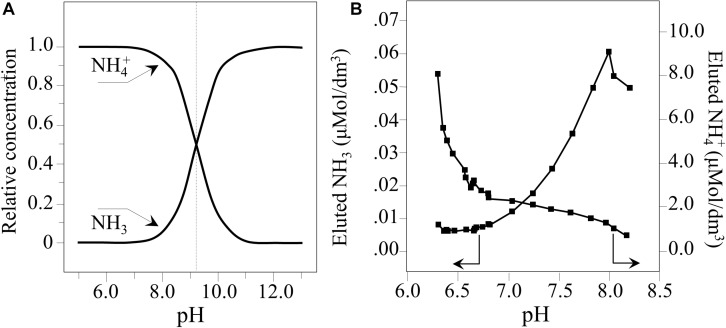
**(A)** Plot of Eq. (4) representing the relative concentrations of NH_3_ and NH_4_^+^. **(B)** Quantitative plots of nitrogen species eluted in water as a function of pH (computed from data in Figure 1A, Eq. 4, and according to Pezzotti et al., 2018).

This indicates that the relative fraction of free NH_3_ at physiological pH is quite low (i.e., 1–2% of the overall amount of ammonium species eluted). However, at a pH of ∼8.3, it increases up to ∼7%. Figure 8A provides a graph of Eq. 4. It shows the relative concentrations of NH_3_ and NH_4_^+^. In Figure 8B, quantitative plots as a function of pH are shown for their concentrations in pure water. These values were computed from the data in Figure 1A using Eq. 4 and calibrated according to published colorimetric ammonia assays (Pezzotti, 2019). Ammonia can readily penetrate the pathogen’s cellular membrane where it cleaves the phosphate deoxyribose DNA backbone (referred to as genome cleavage by alkaline transesterification) (Decrey et al., 2015). However, another important aspect of Si_3_N_4_ in water is the formation of free radicals. This involves a series of transient off-stoichiometric reactions which begins with the breakage of Si-N bonds, the release of free-electrons, and formation of oxygen radicals, as follows (Mezzasalma and Baldovino, 1996; Sonnefeld, 1996; Kajdas, 2013):

(5) Si-N→Si++N+•e-

(6) HO2+e→-H+•OH-

(7) O1+2e→-O•-2

(8) O+2•-H→+HO•-2

(9) Si-OH→2+(s)Si-OH+(s)H(aq)+

(10) Si-OH→(s)Si-O+-(s)H)+(aq

(11) Si-NH→3+(s)Si-NH+2(s)H)+(aq

These reactions (Eqs 5–11) represent a cascade of chemical events which includes free-electron release (Eq. 5), splitting of water molecules (Eq. 6), and the formation of radical oxygen anions and highly oxidative protonated species (Eqs 7, 8). These latter species contribute to the dissociation of surface silanols (Eqs. 9–11), which in turn leads to the formation of additional oxygen radicals, i.e., (≡Si – O^⋅^) and (≡Si – O_2_^⋅−^). Free-electrons also oxidize ammonia (NH_3_) into hydroxylamine (NH_2_OH, i.e., ammonia monooxygenase) and its successive reaction with water to form nitrous acid HNO_2_ with the production of additional free-electrons and protons (Arp and Stein, 2003; Caranto and Lancaster, 2017):

(12) NH+32e+-2H+O→2NHOH2+HO2→HNO+24e+-4H+

(13) NHOH2→NO+3H++3e-

(14) 2HNO→2NO+NO+2-HO2

Equation (12) (i.e., ammonia monooxygenase) provides the free-electrons needed to catalyze NH_3_ oxidation, along with the formation of nitrous acid, additional free-electrons, and hydrogen protons. Equation (13) (i.e., hydroxylamine oxidoreductase) produces nitric oxide (NO), additional free-electrons, and hydrogen protons. The formation of additional NO and nitrite (NO_2_^–^) according to Eq. 14, together with oxygen radicals (O_2_^⋅–^) from Eq. 7 leads to the formation of peroxynitrite, ONOO^–^, as follows (Squadrito and Pryor, 1995; Whittaker et al., 2000):

(15) O+2•-NO•→OO--N=O

This ultimately leads to the formation of nitric oxide (NO) and peroxynitrite (OONO^–^) radicals, which are among the most lethal agents to pathogens (Fang, 1997). The formation of peroxynitrite has been experimentally confirmed in a recent study of the interaction of Si_3_N_4_ and *Candida albicans* using stimulated emission depletion microscopy and a specific fluorescent stain kit for nitrative stress sensing targeting peroxynitrite (Pezzotti, under review). Conversely, peroxynitrite is not toxic to plant cells (Leitner et al., 2009) and NO is a crucial signal in induction of plant resistance against pathogen infections, therefore exerting a positive indirect effect on plant expression of defense-related genes (Vandelle and Delledonne, 2011).

By direct observation, this study confirmed the robust pH buffering of Si_3_N_4_ in an aqueous suspension and the release of gaseous ammonia (Figure 1). The observed pH buffering was a transient phenomenon because of the gradual coverage of the Si_3_N_4_ surface by orthosilicic acid and due to gaseous nitrogen leaving the open system (*cf*. gas bubbles observed in Figure 1D).

In Figures 2D–F, Si_3_N_4_ granules appeared to be electrostatically attracted to the walls of the sporangia, as recently shown also with the viral particles of SARS-CoV-2, as a “catch-and-kill” mechanism (Pezzotti et al., 2020). The cell walls of *Peronosporales* consist of only limited amounts of chitin and predominantly of glucan complexes and mannoproteins (Werner et al., 2002). The latter constituents are linked to β-glucans via glycophosphate groups containing five mannose residues. Phosphorylated mannosyl side chains confer a negative charge to cell walls. Moreover, the functional groups at the surface of the sporangium (i.e., phosphate, carboxyl, and amino groups) become deprotonated in the highly alkaline environment. They interact with positively charged sites on the Si_3_N_4_ surface, which include nitrogen vacancies (charged 3 +) and N-N bonds (charged +) (Pezzotti et al., 2016). Once in contact, the interaction between sporangia and Si_3_N_4_ granules is strongly affected by the highly alkaline pH, which is locally developed, and by the passive diffusion of highly volatile NH_3_ molecules across the cell walls (MacMillan, 1956).

### Interpretation of the Raman Analyses

**FIGURE 9:**
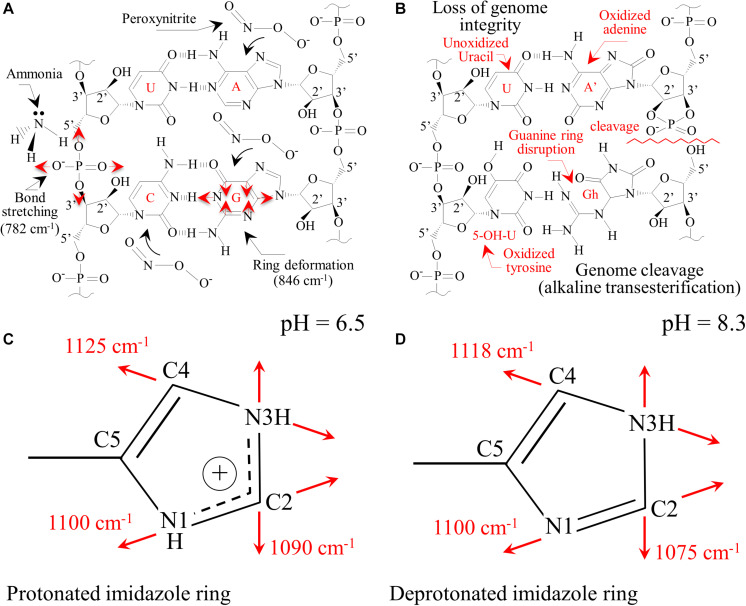
**(A)** Pristine structure of DNA nucleobases for sporangia exposed to water with the main vibrational modes observed; **(B)**: loss of genome integrity due to the presence of passively penetrated NH_3_. Protonation of histidine residues in peptides as a consequence of the alkaline pH burst induced by the dissolution of Si_3_N_4_ in water with the aromatic imidazole ring positively charged **(C)** with both the N atoms in the ring being protonated (pH = 6.5) and in neutral form **(D)** with one proton lost (pH = 8.3).

The main chemical reaction expected by ammonia on nucleic acid is hydrolysis (Jones and Germann, 1916). Nucleic acid is first decomposed into two dinucleotides, one containing adenine and uracil groups, while the other retains guanine and cytosine groups. Although the adenine-uracil dinucleotide is comparatively more stable than the guanine-cytosine, both decompose into mononucleotides at pH values > 8. In the presence of NH_3_, adenine and guanine, and the phosphodiester bonds are deprotonated and strongly destabilized. At any alkaline pH, the hydrogen at N(3) in thymine is also removed due to the weak basicity of the nitrogen ring. Upon exposure to Si_3_N_4_, the most striking spectral variations were the disappearance of the two strongest signals, namely Band 25 and 31 (i.e., related to C’5-O-P-O-C’3 phosphodiester symmetric stretching in DNA and C4-N9-C8 + N1-C2-N3 in-plane deformation of guanine rings, respectively). A significant decrease in intensity, if not the disappearance, of several bands related to adenine (Bands 5, 12, and 36) and cytosine (Bands 6, 10, 11, 19, and 26) was noticed (Figures 7A,B). These observations are in line with previous studies on interactions between Si_3_N_4_ and pathogens (Pezzotti, 2018, 2019). Schematic diagrams of the DNA nucleobases before and after the oxidizing effect created by diffusion of NH_3_ and the nitrogen-free radical reactions (Pezzotti, 2018, 2019) are provided in Figures 9A,B, respectively. The most striking features are the cleavage of the phosphodiester bond in the DNA with the disappearance of stretching, Band 25, the opening of the guanine ring (G→Gh) due to interaction with peroxynitrite radicals, and the disappearance of its strongest ring vibration, Band 31. The significant intensity reduction of Band 14, also contributed by ring breathing mode in guanine, confirms the Gh mechanism shown in Figure 9B. A modification was also observed for the adenine unit A, with the oxidation of its structure (A’). Adenosine oxidation is believed to be responsible for the disappearance of the two ring-deformation Bands 5 and 12, due to the formation of two oxygen double bonds as shown in Figure 9B. The modification of cytosine nucleobase C into the 5-OH-U structure can explain the disappearance of Bands 6 and 10. The former, which in the original cytosine structure represents N3 = C4-N4 and C-C = C bending modes, disappeared because these bonds are replaced with N3-C = O and C-C-OH bonds, respectively, in the oxidized 5-OH-U unit, and the latter, which is from C2 = O bending in the original cytosine structure. It was canceled out by the appearance of a symmetric C4 = O bond in the oxidized 5-OH-U structure. Bands from thymine and uracil (U) appear more stable because they possess only one C = C double bond. Finally, note that some bands related to oxidized nucleobases showed a lower decrease in intensity (i.e., Bands 7, 17, and 21 at 558, 681, and 731 cm^–1^, respectively). However, these bands shared the characteristic of being contributed by lipids (cholesterol, ceramide, and phosphatidylserine, respectively).

Sonois et al. (2008) described the Raman behavior of several amino acids by both experiments and theoretical calculations. In the case of histidine, environments with increasing pH led to the appearance of new Raman bands at ∼613, 656, and 860 cm^–1^. These three bands could likely correspond to the new bands detected in sporangia exposed to Si_3_N_4_ (Figure 7B) and labeled as Bands 11^∗^, 15^∗^, and 32^∗^. The side chain of a histidine molecule is an aromatic imidazole ring that contains 6 π–electrons. Depending on environmental pH, different tautomeric and ionic forms of histidine can be present. At pH < 6, a positively charged form dominates with both the N atoms in the protonated ring (Figure 9C). Conversely, at increasing pH-values, histidine loses one proton in its imidazole ring, which gradually gives rise to neutral forms (Figure 9D). Valery et al. (2015) studied the conformational change of self-assembled histidine-containing peptides and their stabilized globular conformation at high pH. Vibrational spectroscopy assessments revealed histidine-serine H-bond and histidine-aromatic interactions. At pH = 8.5, histidine deprotonation occurred and altered the C-N ring stretching bands in the Raman spectrum in the frequency range 1,050∼1,150 cm^–1^.

**FIGURE 10:**
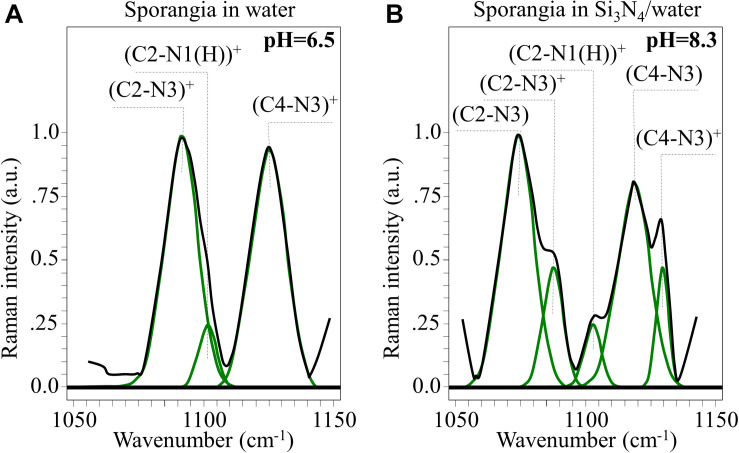
In **(A,B)**, high resolution Raman signals from the spectral zone 1,050∼1,150 cm^– 1^, related to the vibrations of charged and neutral imidazole ring (*cf*. structures in Figures 9C,D, respectively).

In an attempt to strengthen the histidine interpretation for the newly formed Bands 11^∗^, 15^∗^, and 32^∗^, we monitored the C-N stretching spectral area for the imidazole ring in the frequency range 1,050∼1,150 cm^–1^. Different trends were observed when comparing Raman spectra collected on sporangia in pure water (pH = 6.5) versus the Si_3_N_4_ suspension (pH = 8.3, Figures 10A,B, respectively). According to a recent paper by Pogostin et al., 2019 and considering the ring structures depicted in Figures 9C,D, the spectroscopic fingerprints for deprotonation of the histidine ring are as follows:

(i) A clear new shoulder Band appeared at ∼1,075 cm^–1^. This is in addition to the band originally observed at ∼1,090 cm^–1^, whose intensity was significantly weaker. These bands are related to stretching vibrations of the (C2-N3)^+^ and (C2-N3) bond configurations, respectively. The former configuration involves a stronger bond (i.e., due to N1-C2-N3 electron sharing). Its vibrational energy is greater and, thus, it appears at higher frequencies.
(ii) A similar trend was observed for the stretching bands in the pristine (C4-N3)^+^ and deprotonated (C4-N3) bond configurations, which appeared at ∼1,125 (shoulder band) and ∼1,118 cm^–1^ (pristine band), respectively. This trend can be explained using the same reasoning given in (i), even though the frequency shift toward a lower frequency is less pronounced than in the case of (i). This circumstance is related to the balance of bonding strength within the deprotonated ring. The C2-N3 bond is weaker than the C4-N3 bond because its neighboring double bond N1 = C2 is stronger than the double bond C4 = C5 next to C4-N3 (i.e., due to the higher electronegativity of N over C).
(iii) No significant shift or intensity variation, but only a slight broadening, was observed for the stretching band related to the (C2-N1(H))^+^ bond (at ∼1,100 cm^–1^) when the ring configuration was deprotonated.

Additional Raman analyses of the imidazole ring of histidine residues in the C = C and C = N spectral zone at ∼1,600 cm^–1^ (not shown) provided features that were consistent with the results shown in Figure 10. Based on the above Raman analyses, the Raman spectroscopic signatures are related to deprotonation occurring in a highly alkaline environment. These reactions are common to histidine-containing peptides at high pH-values.

Histidine kinase proteins are present in most prokaryotic and eukaryotic organisms. They regulate several adaptive transcriptional responses to a variety of environmental factors. In oomycetes, functional analyses of histidine kinases are missing, while phosphorylation at histidine sites is a common metabolic response of fungi to osmotic stress (Paithoonrangsarid et al., 2004; Herivaux et al., 2016). In response to perceiving osmotic stress as a change in environmental conditions, conserved histidine residues are phosphorylated with a phosphate group from adenosine triphosphate (Paithoonrangsarid et al., 2004), which agrees with the reduction in adenosine Raman bands detected in Figure 7B. Successive transfer of the phosphoryl group to conserved aspartate residues results in a modulation that mediates signal transfer to the signaling pathway (Paithoonrangsarid et al., 2004). Because of the highly localized alkalinity between Si_3_N_4_ granules and sporangial walls, an appreciable fraction of NH_3_ can penetrate the cell and severely alters its osmotic balance. Therefore, alteration of the Raman data due to Si_3_N_4_ exposure suggests that sporangia reacted to osmotic stress; and the histidine kinase perceived osmotic stress similar to what was hypothesized for *Saccharomyces cerevisiae* (Paithoonrangsarid et al., 2004). It should be noted that in the model oomycete *Phytophthora infestans*, protein kinases have been found to be involved with sporangial cleavage during germination (Judelson and Robert, 2002).

In the present study, the hypothesis that the variations observed in the Raman spectrum of sporangia exposed to Si_3_N_4_ may partly arise from a metabolic reaction of the pathogen to environmental stress is corroborated by the disappearance of a main signal (i.e., Band 33 at 893 cm^–1^) and by a significant reduction in the intensity of all other signals (i.e., Bands 3, 14, 15, and 19 at 500, 643, 649, and 710 cm^–1^, respectively) related to cellulose (and/or chitin) and other linear carbohydrates in the structure of the cell walls. Linear polymeric chains in cellulose are linked together by β-glycosidic bonds. These bonds are not affected by the alkaline pH levels induced by Si_3_N_4_, or by any direct interaction with NH_3_. On the other hand, hydrolytic enzymes can break down the glycosidic bonds and thereby alter the cell walls of phytopathogens (Judelson and Robert, 2002). Given how the Raman experiments were conducted, the enzymatic reaction could only be intrinsic to sporangia themselves. Moreover, the wall composition needs to be remodeled during sporangial germination and dissolution of sporangial papilla (Judelson and Robert, 2002; Ene et al., 2015).

Turchini et al., 2000 measured a 50% decrease in chitin content for fungal cells grown in a high-osmolarity medium as compared to those grown in low-osmolarity, in agreement with previous data showing that the chitin synthase activity in fungi is higher for cells grown in a low- vs. a high-osmolarity media (Gooday and Shofield, 1995; Deshpande et al., 1997). These researchers interpreted the observed weakening of the fungal walls in high-osmolarity medium as a rescuing mechanism to enable membrane stretching and enhance the probability of maintaining cell integrity. In analogy with fungi, the disappearance of the main Raman signal from cell wall (Band 33) for sporangia exposed to Si_3_N_4_ could be interpreted as an enzymatic fingerprint activated by the cells in the attempt to resist osmotic stress.

### Si_3_N_4_ in the Context of Low-Impact Defense Against Grapevine Downy Mildew

Multiple chemical treatments are currently used in every growing season to control *P. viticola*, largely contributing to the environmental impact of grapevine cultivation worldwide. Consumer concerns and legislative restriction have strongly limited the number and type of chemicals that can be applied and a big research effort is put in finding alternative solutions (Dagostin et al., 2011; Pertot et al., 2017). Three strategies have so far been pursued to replace/reduce the use of conventional agrochemicals in organic agriculture: (i) antagonist biocontrol agents (Perazzolli et al., 2008); (ii) biological or chemical inducers of plant resistance responses (Aziz et al., 2003, 2006; Hadrami et al., 2010; El-Naggar et al., 2012; Garde-Cerdan et al., 2017); and, (iii) biopesticides from natural sources, such as plant extracts or inorganic salts (Deliopoulos et al., 2010; Dagostin et al., 2011; Lukas et al., 2016).

While the identification and use of microorganisms as direct *Plasmopara* antagonists is yet very limited (Puopolo et al., 2014) and has not reached commercial applications yet, different natural compounds have been tested as eco-friendly formulations against *P. viticola*. The naturally occurring oligosaccharide chitosan and the water-soluble β-1,3-glucan laminarin obtained from the brown alga *Laminaria digitata* are used as efficient inducers of plant defense reactions and accumulation of antimicrobial phytoalexins (Aziz et al., 2003, 2006; Hadrami et al., 2010). However, some undesired effects of these polysaccharides on the amino acid composition of must from grapevines have been reported, that may affect the final wine quality (Garde-Cerdan et al., 2017).

Other naturally derived compounds tested include hydrolyzed proteins, plant extracts, and inorganic salts. In this context, a recent promising strategy has been proposed based on the use of a selected aptamer peptide, specifically inhibiting *P. viticola* cellulose synthase 2, and therefore preventing infection with no adverse effects on non-target organisms (Colombo et al., 2015, 2020).

**FIGURE 11:**
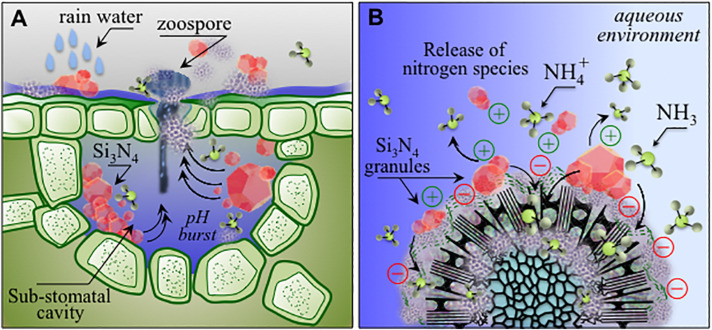
**(A)** Schematic drafts of pH buffering effect and related ammonia elution in stomata entrapping micrometer-sized Si_3_N_4_ granules upon rain; **(B)**: charge attraction of Si_3_N_4_ granules to sporangia with the formation of ammonium and ammonia as antimicrobial agents active at the pathogen/Si_3_N_4_ interface.

Among inorganic salts, examples include mainly bicarbonates, phosphates, silicates, chlorides and phosphites (Deliopoulos et al., 2010). Their activity has been mainly reported against powdery mildews of different crops including grapevine, while only sodium bicarbonate showed a limited efficacy against grapevine downy mildew (Lukas et al., 2016). The development of silicon nitride-based phytosanitary products falls within this last category. In the present context, silicates deserve a particular mention: several soluble silicate salts possess direct and indirect activities against different fungal infections, acting by both stimulation of the plant’s natural defense mechanisms and strengthening of plant cell walls (Deliopoulos et al., 2010). The production of SiO_2_ and Si(OH)_4_ from Si_3_N_4_ (as described in reactions 1–3) may thus complement the direct action of ammonia on *P. viticola* sporangia and zoospores by inducing plant resistance, which can be at least partially responsible for the almost complete inhibition of the infection process observed in our experiments. Moreover, in comparison to soluble salts, which are readily washed off by rain, Si_3_N_4_ could provide a more lasting protection through different elution cycles of ammonium moieties from the insoluble powder, and generation of reactive nitrogen species, in line with previous studies on human pathogens (Wink et al., 1991; Hetrick et al., 2008; Schairer et al., 2012). In fact, upon treatment, Si_3_N_4_ particles may remain trapped inside the stomata (Figure 11A), and during rain events water may repeatedly generating a hostile chemical hostile environment (Figure 11B), possibly impairing sporangia emission in secondary infection cycles, thus reducing the need for further chemical treatments. Si_3_N_4_ is a biocompatible ceramic not toxic to human cells, widely used as an implantable biomaterial with antibacterial properties, containing only environmentally friendly elements, intrinsic to the earth’s prehistory (Riley, 2000). Its applications are expected to extend beyond the medical and engineering fields due to its multiple properties and low cost. The multi-mechanistic lytic reactions occurring in the pathogen’s cytoplasm due to the diffusion of NH_3_ across cell walls reduces the probability of selection of adaptive mutations in the pathogen population. Ammonium, which is generated by Si_3_N_4_ decomposition, is the primary inorganic species involved in the synthesis of organic nitrogen. Ammonium and nitrate ions in the soil are directly absorbed through root-specific transporters and effectively utilized (Glass et al., 2002). In the case of grapevines, NH_4_^+^ represents up to 80% of the total nitrogen before veraison while decreasing to 5–10% after maturation and even lower after must fermentation (Martins et al., 2012). Nitrogen eluted from Si_3_N_4_ may contribute to an improvement in berry quality and fermentation conditions. As a limiting factor, care should be taken to balance the quantities of nitrogen from fertilizers and Si_3_N_4_ because excess nitrogen may alter the production of phenolic compounds and the taste or quality of both grapes and wine (Waters et al., 1998). Several plant species benefit from Si fertilization, particularly in alleviating biotic and abiotic stresses (Fauteux et al., 2005, 2006).

## Conclusion

This study provides new insight into the effect of Si_3_N_4_ against grapevine infections by *P. viticola*. As an inorganic environmentally friendly agent, it has the potential to replace heavy metal agrochemicals and to be included among newer antipathogenic molecules. The unique chemistry of Si_3_N_4_ induces osmotic stress in sporangia and zoospores and prevents infections even at concentrations as low as 1.5 vol.%, which is in the molar range of concentration used for other inorganic salts in agriculture application, such as bicarbonates (Deliopoulos et al., 2010). Raman experiments provided information on chemical mechanisms, which included cleavage of phosphate deoxyribose backbone and disruption of guanine rings. Experiments on leaves on different grapevine varieties showed that Si_3_N_4_ was effective in severely reducing or blocking the infection process at very early stages, affecting sporangia germination and zoospores viability, as revealed by microscopic observations. In conclusion, Si_3_N_4_ can be regarded as a promising biopesticide with multiple benefits in comparison to conventional synthetic products and technical advantages over other inorganic salts and could therefore be developed as a useful component in integrated disease management. Eventually, its actual exploitation will need additional validations, namely field experiments and evaluation of plant’s fitness after multiple application of Si3N4.

## Data Availability Statement

The raw data supporting the conclusions of this article will be made available by the authors, without undue reservation.

## Author Contributions

GP and AP were responsible of conceptualization and writing. YF, FB, EM, EV, and MG performed the experiments. BM and SB produced and provided the silicon nitride powders for the experiments. WZ and KM took care of data interpretation and analysis. All authors contributed to the article and approved the submitted version.

## Conflict of Interest

BM and SB are principals and employees of SINTX Technologies, Inc. GP has been a consultant and member of the Scientific Advisory Board of SINTX Technologies, Inc., in the early stage of this work. The remaining authors declare that the research was conducted in the absence of any commercial or financial relationships that could be construed as a potential conflict of interest.

## References

[B1] Adl S. M. Bass D. Lane C. E. Lukes J. Schoch C. L. Smirnov A. (2019). Revisions to the classification, nomenclature, and diversity of Eukaryotes. *J. Eukaryot. Microbiol.* 6 4–119. 10.1111/jeu.12691 PMC6492006 30257078

[B2] Armijo G. Schlechter R. Agurto M. Muñoz D. Nuñes C. Arce-Johnson P. (2016). Grapevine pathogenic microorganisms: understanding infection strategies and host response scenarios. *Front. Plant Sci.* 7:382. 10.3389/fpls.2016.00382 27066032 PMC4811896

[B3] Arp D. J. Stein L. Y. (2003). Metabolism of inorganic N compounds by ammonia-oxidizing bacteria. *Crit. Rev. Biochem. Mol. Biol.* 38 471–495. 10.1080/10409230390267446 14695127

[B4] Aziz A. Poinssot B. Daire X. Adrian M. Bezìier A. Lambert B. (2003). Laminarin elicits defense responses in grapevine and induces protection against *Botrytis cinerea* and *Plasmopara viticola*. *Mol. Plant Microbe Interact.* 16 1118–1128. 10.1094/mpmi.2003.16.12.1118 14651345

[B5] Aziz A. Trotel-Aziz P. Dhuicq L. Jeandet P. Couderchet M. Vernet G. (2006). Chitosan oligomers and copper sulfate induce grapevine defense reactions and resistance to gray mold and downy mildew. *Phytopathology* 96 1188–1194. 10.1094/phyto-96-1188 18943955

[B6] Burruano S. (2000). The life-cycle of *Plasmopara viticola*, cause of downy mildew of vine. *Mycologist* 14 179–182. 10.1016/s0269-915x(00)80040-3

[B7] Cael J. J. Koenig J. L. Blackwell J. (1974). Infrared and Raman spectroscopy of carbohydrates: part IV. Identification of configuration- and conformation-sensitive modes for D-glucose by normal coordinate analysis. *Carbohydr. Res.* 32 79–91.

[B8] Caffi T. Rossi V. Bugiani R. (2010). Evaluation of a warning system for controlling primary infections of grapevine downy mildew. *Plant Dis.* 94 709–716. 10.1094/pdis-94-6-0709 30754303

[B9] Calvert G. C. VanBuren Huffmon G. III Rambo W. M. Jr. Smith M. W. McEntire B. J. Bal B. S. (2020). Clinical outcomes for lumbar fusion using silicon nitride versus other biomaterials. *J. Spine Surg.* 6 33–48. 10.21037/jss.2019.12.11 32309644 PMC7154368

[B10] Caranto J. D. Lancaster K. M. (2017). Nitric oxide is an obligate bacterial nitrification intermediate produced by hydroxylamine oxidoreductase. *Proc. Natl. Acad. Sci. U.S.A.* 114 8217–8222. 10.1073/pnas.1704504114 28716929 PMC5547625

[B11] Colombo M. Masiero S. Rosa S. Caporali E. Toffolatti S. L. Mizzotti C. (2020). NoPv1: a synthetic antimicrobial peptide aptamer targeting the causal agents of grapevine downy mildew and potato late blight. *Sci. Rep.* 10:17574. 10.1038/s41598-020-73027-x PMC7567880 33067553

[B12] Colombo M. Mizzotti C. Masiero S. Kater M. M. Pesaresi P. (2015). Peptide aptamers: the versatile role of specific protein function inhibitors in plant biotechnology. *J. Integr. Plant Biol.* 57 892–901. 10.1111/jipb.12368 25966787

[B13] Corbett E. C. Zichy V. Goral J. Passingham C. (1991). Fourier transform Raman studies of materials and compounds of biological importance - II. The effect of moisture on the molecular structure of the alpha and beta anomers of D-glucose. *Spectrochim. Acta A Mol. Spectrosc.* 47 1399–1411. 10.1016/0584-8539(91)80231-7

[B14] Czamara K. Majzner K. Pacia M. Z. Kochan K. Kaczor A. Baranska M. (2015). Raman spectroscopy of lipids: a review. *J. Raman Spectrosc.* 46 4–20. 10.1002/jrs.4607

[B15] Dagostin S. Schärer H. J. Pertot I. Tamm L. (2011). Are there alternatives to copper for controlling grapevine downy mildew in organic viticulture. *Crop Prot.* 30 776–788. 10.1016/j.cropro.2011.02.031

[B16] Dahlin P. Srivastava V. Ekengren S. McKee L. S. Bulone V. (2017). Comparative analysis of sterol acquisition in the oomycetes *Saprolegnia parasitica* and *Phytophthora infestans*. *PLoS One* 12:e0170873. 10.1371/journal.pone.0170873 28152045 PMC5289490

[B17] Dai Y. Guo H. Chu L. He Z. Wang M. Zhang S. (2019). Promoting osteoblasts responses in vitro and improving osteointegration in vivo through bioactive coating of nanosilicon nitride on polyetheretherketone. *J. Orthop. Translat.* 24 198–208. 10.1016/j.jot.2019.10.011 33101971 PMC7548345

[B18] De Gussem K. Vandenabeele P. Verbeken A. Moens L. (2005). Raman spectroscopic study of *Lactarius* spores (Russulales, Fungi). *Spectrochim. Acta A Mol. Biomol. Spectrosc.* 61 2896–2908. 10.1016/j.saa.2004.10.038 16165029

[B19] Decrey L. Kazama S. Udert K. M. Kohn T. (2015). Ammonia as an in situ sanitizer: inactivation kinetics and mechanisms of the ssRNA virus MS2 by NH3. *Environ. Sci. Technol.* 49 1060–1067. 10.1021/es5044529 25496714

[B20] Deliopoulos T. Kettlewell P. S. Hare M. C. (2010). Fungal disease suppression by inorganic salts: a review. *Crop Prot.* 29 1059–1065. 10.1016/j.cropro.2010.05.011

[B21] Deshpande M. O’Donnell R. Gooday G. W. (1997). Regulation of chitin synthase activity in the dimorphic fungus *Benjaminiella poitrasii* by external osmotic pressure. *FEMS Microbiol. Lett.* 152 327–332. 10.1111/j.1574-6968.1997.tb10447.x 9231427

[B22] Elliot C. G. (1983). “Physiology of sexual reproduction in *Phytophthora*,” in *Phytophthora: Its Biology, Taxonomy, Ecology and Pathology*, eds Erwin D. C. Bartnicki-Garcia S. Tsao P. G. (Saint Paul, MN: The American Phytopathological Society), 71–80.

[B23] El-Naggar M. A. Alkahtani M. D. F. Yassin M. A. Morsy K. M. (2012). New approach to acquired resistance enhancement against *Plasmopara viticola* using different biotic inducers. *J. Plant Sci.* 7 67–77. 10.3923/jps.2012.67.77

[B24] Ene I. V. Walker L. A. Schiavone M. Lee K. K. Martin-Yken H. Dague E. (2015). Cell wall remodeling enzymes modulate cell wall elasticity and osmotic stress resistance. *mBio* 6:e00986-15. 10.1128/mBio.00986-15 PMC4551979 26220968

[B25] Fang F. C. (1997). Perspectives series: host/pathogen interactions. Mechanisms of nitric oxide-related antimicrobial activity. *J. Clin. Invest.* 99 2818–2825. 10.1172/jci119473 9185502 PMC508130

[B26] Fauteux F. Chain F. Belzile F. Menzies J. G. Bélanger R. R. (2006). The protective role of silicon in the *Arabidopsis*–powdery mildew pathosystem. *Proc. Natl. Acad. Sci. U.S.A.* 103 17554–17559. 10.1073/pnas.0606330103 17082308 PMC1859967

[B27] Fauteux F. Rémus-Borel W. Menzies J. G. Bélanger R. R. (2005). Silicon and plant disease resistance against pathogenic fungi. *FEMS Microbiol. Lett.* 249 1–6. 10.1016/j.femsle.2005.06.034 16006059

[B28] Gamm M. Heloir M. C. Adriam M. (2015). Trehalose and trehalose-6-phosphate induce stomatal movements and interfere with ABA-induced stomatal closure in grapevine. *OENO One* 49 165–171. 10.20870/oeno-one.2015.49.3.84

[B29] Garde-Cerdan T. Mancini V. Carrasco-Quiroz M. Servili A. Gutierrez-Gamboa G. Foglia R. (2017). Chitosan and laminarin as alternatives to copper for *Plasmopara viticola* control: effect on grape amino acid. *J. Agric. Food Chem.* 65 7379–7386. 10.1021/acs.jafc.7b02352 28759217

[B30] Gessler C. Pertot I. Perazzolli M. (2011). *Plasmopara viticola*: a review of knowledge on downy mildew of grapevine and effective disease management. *Phytopatol. Mediterr.* 50 2–44.

[B31] Glass A. D. M. Britto D. T. Kaiser B. N. Kinghorn J. R. Kronzucker H. J. Kumar A. (2002). The regulation of nitrate and ammonium transport systems in plants. *J. Exp. Bot.* 53 855–864. 10.1093/jexbot/53.370.855 11912228

[B32] Gooday G. Shofield D. A. (1995). Regulation of chitin synthesis during growth of fungal hyphae: the possible participation of membrane stress. *Can. J. Bot.* 73 S114–S121.

[B33] Hadrami A. E. Adam L. R. Hadrami I. E. Fouad D. (2010). Chitosan in plant protection. *Mar. Drugs* 8 968–987. 20479963 10.3390/md8040968 PMC2866471

[B34] Herivaux A. So Y. S. Gastebois A. Latge J. P. Bouchara J. P. Bahn Y. S. (2016). Major sensing proteins in pathogenic fungi: the hybrid histidine kinase family. *PLoS Pathog.* 12:e1005683. 10.1371/journal.ppat.1005683 27467512 PMC4965123

[B35] Hetrick E. M. Shin J. H. Stasko N. A. Johnson C. B. Wespe D. A. Holmuhamedov E. (2008). Bactericidal efficacy of nitric oxide-releasing silica nanoparticles. *ACS Nano* 2 235–246. 19206623 10.1021/nn700191f PMC3571086

[B36] Jones W. Germann H. C. (1916). Hydrolysis of yeast nucleic acid with ammonia. *J. Biol. Chem.* 25 93–102.

[B37] Judelson H. S. Robert S. (2002). Novel protein kinase induced during sporangial cleavage in the oomycete *Phytophthora infestans*. *Eukaryot. Cell* 1 687–695. 10.1128/ec.1.5.687-695.2002 12455688 PMC126747

[B38] Kajdas C. (2013). *General Approach to Mechanochemistry and Its Relation to Tribochemistry.* London: IntechOpen, 209–240.

[B39] Kiefer B. Riemann M. Büche C. Kassemeyer H. H. Nick P. (2002). The host guides morphogenesis and stomatal targeting in the grapevine pathogen *Plasmopara viticola*. *Planta* 215 387–393. 10.1007/s00425-002-0760-2 12111219

[B40] Krafft C. Neudert L. Simat T. Salzer R. (2005). Near infrared Raman spectra of human brain lipids. *Spectrochim. Acta A Mol. Spectrosc.* 61 1529–1535. 10.1016/j.saa.2004.11.017 15820887

[B41] Laarz E. Zhmud B. V. Bergstroem L. (2000). Dissolution and deagglomeration of silicon nitride in aqueous medium. *J. Am. Ceram. Soc.* 83 2394–2400. 10.1111/j.1151-2916.2000.tb01567.x

[B42] Leitner M. Vandelle E. Gaupels F. Bellin D. Delledonne M. (2009). NO signals in the haze: nitric oxide signalling in plant defence. *Curr. Opin. Plant Biol.* 12 451–458. 10.1016/j.pbi.2009.05.012 19608448

[B43] Lopes R. P. Marques M. P. M. Valero R. Tomkinson J. Batista de Carvalho L. A. E. (2012). Guanine: a combined study using vibrational spectroscopy and theoretical methods. *Spectrosc. Int. J.* 27 273–292. 10.1155/2012/168286

[B44] Lopes R. P. Valero R. Tomkinson J. Marques M. P. M. Batista de Carvalho L. A. E. (2013). Applying vibrational spectroscopy to the study of nucleobases – adenine as a case study. *New J. Chem.* 37 2691–2699. 10.1039/c3nj00445g

[B45] Lu H. Zhu Z. Y. Dong L. L. Jia X. M. Sun X. R. Yan L. (2011). Lack of trehalose accelerates H2O2-induced *Candida albicans* apoptosis through regulating Ca2+ signaling pathway and caspase activity. *PLoS One* 6:e15808. 10.1371/journal.pone.0015808 21246042 PMC3016397

[B46] Lukas K. Innerebner G. Kelderer M. Finckh M. R. Hohmann P. (2016). Efficacy of copper alternatives applied as stop-sprays against *Plasmopara viticola* in grapevine. *J. Plant Dis. Prot.* 123 171–176. 10.1007/s41348-016-0024-1

[B47] Machovic V. Lapcak L. Havelcova M. Borecka L. Novotna M. Novotna M. (2017). Analysis of European honeybee (*Apis mellifera*) wings using ATR-FTIR and Raman spectroscopy: a pilot study. *Sci. Agric. Bohem.* 48 22–29. 10.1515/sab-2017-0004

[B48] MacMillan A. (1956). The entry of ammonia into fungal cells. *J. Exp. Bot.* 19 113–126. 10.1093/jxb/7.1.113 12432039

[B49] Madzharova F. Heiner Z. Guehlke M. Kneipp J. (2016). Surface-enhanced hyper-Raman spectra of adenine, guanine, cytosine, thymine, and uracil. *J. Phys. Chem. C* 120 15415–15423. 10.1021/acs.jpcc.6b02753 28077982 PMC5215682

[B50] Martinez-Prada I. Kortekamp A. (2015). Effect of sterol biosynthesis inhibitors and azole-type inducers on growth and development of *Plasmopara viticola* on grapevine. *Vitis* 54 91–96.

[B51] Martins V. Cunha A. Geros H. Hanana M. Blumwald E. (2012). “Mineral compounds in the grape berry,” in *The Biochemistry of Grape Berry*, eds Geros H. Chaves M. Delrot S. (Sharjah: Bentham Science Publishers), 23–43. 10.2174/978160805360511201010023

[B52] Mathlouthi M. Seuvre A. M. (1984). F.T.-I.R. and laser-Raman spectra of thymine and thymidine. *Carbohydr. Res.* 134 23–38. 10.1016/0008-6215(84)85019-3

[B53] Mathlouthi M. Seuvre A. M. (1986a). F.T.-I.R. and laser-Raman spectra of cytosine and cytidine. *Carbohydr. Res.* 146 1–13. 10.1016/0008-6215(86)85019-4 3955567

[B54] Mathlouthi M. Seuvre A. M. (1986b). F.T.-I.R. and laser-Raman spectra of guanine and guanosine. *Carbohydr. Res.* 146 15–27. 10.1016/0008-6215(86)85020-0 3955569

[B55] Melida H. Sandoval-Sierra J. V. Dieguez-Uribeondo J. Bulone V. (2013). Analyses of extracellular carbohydrates in oomycetes unveil the existence of three different cell wall types. *Eukaryot. Cell* 12 194–203. 10.1128/ec.00288-12 23204192 PMC3571302

[B56] Mezzasalma S. Baldovino D. (1996). Characterization of silicon nitride surface in water and acid environment: a general approach to the colloidal suspensions. *J. Colloid Interface Sci.* 180 413–420. 10.1006/jcis.1996.0320

[B57] Mishra S. Tandon P. (2012). DFT study of structure and vibrational spectra of ceramide 3: comparison to experimental data. *Mol. Simul.* 38 872–881. 10.1080/08927022.2012.662645 22206896

[B58] Muthmann R. Nardin P. (2007). *The Use of Plant Protection Products in the European Union: Data 1992-2003.* Luxembourg: Office for Official Publications of the European Communities.

[B59] Negrel L. Halter D. Wiedermann-Merdinoglu S. Rustenholz C. Merdinoglu D. Hugueney P. (2018). Identification of lipid markers of *Plasmopara viticola* infection in grapevine using a non-targeted metabolomics approach. *Front. Plant Sci.* 9:360. 10.3389/fpls.2018.00360 29619037 PMC5871909

[B60] Noothalapati H. Sasaki T. Kaino T. Kawamukai M. Ando M. Hamaguchi H.-O. (2016). Label-free chemical imaging of fungal spore walls by Raman microscopy and multivariate curve resolution analysis. *Sci. Rep.* 6:27789. 10.1038/srep27789 PMC4899791 27278218

[B61] Notingher I. Bisson I. Polak J. M. Hench L. L. (2004). *In situ* spectroscopic study of nucleic acids in differentiating embryonic stem cells. *Vib. Spectrosc.* 35 199–203. 10.1016/j.vibspec.2004.01.014

[B62] Paithoonrangsarid K. Shoumskaya M. A. Kanesaki Y. Satoh S. Tabata S. Los D. A. (2004). Five histidine kinases perceive osmotic stress and regulate distinct sets of genes in *Synechocystis*. *J. Biol. Chem.* 279 53078–53086. 10.1074/jbc.m410162200 15471853

[B63] Parker D. R. Norvell W. A. Chaney R. L. (1995). “GEOCHEM-PC – a chemical speciation program for IBM and compatible personal computers,” in *Chemical Equilibria and Reaction Models*, eds Loeppert R. H. Schwab A. D. Goldberg S. (Madison, WI: Soil Science Society of America), 253–269. 10.2136/sssaspecpub42.c13

[B64] Perazzolli M. Dagostin S. Ferrari A. Elad Y. (2008). Induction of systemic resistance against *Plasmopara viticola* in grapevine by *Trichoderma harzianum* T39 and benzothiadiazole. *Biol. Control* 47 228–234. 10.1016/j.biocontrol.2008.08.008

[B65] Pertot I. Caffi T. Rossi V. Mugnai L. Hoffmann C. Grando M. S. (2017). A critical review of plant protection tools for reducing pesticide use on grapevine and new perspectives for the implementation of IPM in viticulture. *Crop Prot.* 97 70–84. 10.1016/j.cropro.2016.11.025

[B66] Pezzotti G. (2018). A spontaneous solid-state NO donor to fight antibiotic resistant bacteria. *Mater. Today Chem.* 9 80–90. 10.1016/j.mtchem.2018.05.004

[B67] Pezzotti G. (2019). Silicon nitride: a bioceramic with a gift. *ACS Appl. Mater. Interfaces* 11 26619–26636. 10.1021/acsami.9b07997 31251018

[B68] Pezzotti G. Bock R. M. McEntire B. J. Adachi T. Marin E. Boschetto F. (2018). *In vitro* antibacterial activity of oxide and non-oxide bioceramics for arthroplastic devices: I. *In situ* time-lapse Raman spectroscopy. *Analyst* 13 3708–3721. 10.1039/c8an00233a 29987284

[B69] Pezzotti G. Marin E. Adachi T. Rondinella A. Boschetto F. Zhu W. (2017). Bioactive silicon nitride: a new therapeutic material for osteoarthropathy. *Sci. Rep.* 7:44848. 10.1038/srep44848 PMC5361106 28327664

[B70] Pezzotti G. McEntire B. J. Bock R. Zhu W. Boschetto F. Rondinella A. (2016). *In situ* spectroscopic screening of osteosarcoma living cells on stoichiometry-modulated silicon nitride bioceramic surfaces. *ACS Biomater. Sci. Eng.* 2 1121–1134. 10.1021/acsbiomaterials.6b00126 33465870

[B71] Pezzotti G. Ohgitani E. Shin-Ya M. Adachi T. Marin E. Boschetto F. (2020). Instantaneous “catch-and-kill” inactivation of SARS-CoV-2 by nitride ceramics. *Clin. Transl. Med.* 10:e212. 10.1002/ctm2.212 33135340 PMC7568850

[B72] Pogostin B. H. Malmendal A. Londergan C. H. Akerfeldt K. S. (2019). pKa determination of a histidine residue in a short peptide using Raman spectroscopy. *Molecules* 24 405–417. 10.3390/molecules24030405 30678032 PMC6385126

[B73] Polesani M. Bortesi L. Ferrarini A. Zamboni A. Fasoli M. Zadra C. (2010). General and species-specific transcriptional responses to downy mildew infection in a susceptible (*Vitis vinifera*) and a resistant (*V. riparia*) grapevine species. *BMC Genomics* 11:117. 10.1186/1471-2164-11-117 20167053 PMC2831845

[B74] Puopolo G. Cimmino A. Palmieri M. C. Giovannini O. Evidente A. Pertot I. (2014). *Lysobacter capsici* AZ78 produces cyclo(L-Pro-L-Tyr), a 2,5-diketopiperazine with toxic activity against sporangia of *Phytophthora infestans* and *Plasmopara viticola*. *J. Appl. Microbiol.* 117 1168–1180. 10.1111/jam.12611 25066530

[B75] Reganold J. P. Wachter J. M. (2016). Organic agriculture in the twenty-first century. *Nat. Plants* 2 15221–15229. 27249193 10.1038/nplants.2015.221

[B76] Ricker K. E. Bostock R. M. (1992). Evidence for release of the elicitor arachidonic acid and its metabolites from sporangia of *Phytophthora infestans* during infection of potato. *Physiol. Mol. Plant Pathol.* 41 61–72. 10.1016/0885-5765(92)90049-2

[B77] Riley F. L. (2000). Silicon nitride and related materials. *J. Am. Ceram. Soc.* 83 245–265. 10.1111/j.1151-2916.2000.tb01182.x

[B78] Sawyer C. N. McCarty P. L. (1978). *Chemistry for Environmental Engineering*, 3rd Edn New York, NY: McGraw-Hill Book Company, 532.

[B79] Schairer D. O. Chouake J. S. Nosanchuk J. D. Friedman A. J. (2012). The potential of nitric oxide releasing therapies as antimicrobial agents. *Virulence* 3 271–279. 10.4161/viru.20328 22546899 PMC3442839

[B80] Schäufele I. Hamm U. (2017). Consumers’ perceptions, preferences and willingness-to-pay for wine and sustainability characteristics: a review. *J. Clean. Prod.* 147 379–394. 10.1016/j.jclepro.2017.01.118

[B81] She C. Y. Dinh N. D. Tu A. T. (1974). Laser Raman scattering of glucosamine, *N*-acetylglucosamine, and glucuronic acid. *Biochim. Biophys. Acta* 372 345–357. 10.1016/0304-4165(74)90196-2

[B82] Sonnefeld J. (1996). Determination of surface charge density parameters of silicon nitride. *Colloids Surf. A Physicochem. Eng. Asp.* 108 27–31. 10.1016/0927-7757(95)03356-4

[B83] Sonois V. Esteve A. Zwick A. Faller P. Bacsa W. (2008). Raman study and DFT calculations of amino acids. *Techconnect Briefs* 1 352–355.

[B84] Squadrito G. L. Pryor W. A. (1995). The formation of peroxynitrite *in vivo* from nitric oxide and superoxide. *Chem. Biol. Interact.* 96 203–206. 10.1016/0009-2797(94)03591-u 7728908

[B85] Steimetz E. Trouvelot S. Gindro K. Bordier A. Poinssot B. Adrian M. (2012). Influence of leaf age on induced resistance in grapevine against *Plasmopara viticola*. *Physiol. Mol. Plant Pathol.* 79 89–96. 10.1016/j.pmpp.2012.05.004

[B86] Töpfer R. Eibach R. (2016). Breeding the next-generation disease-resistant grapevine varieties. *Wine Vitic. J.* 5 41–47.

[B87] Troester V. Setzer T. Hirth T. Pecina A. Kortekamp A. Nick P. (2017). Probing the contractile vacuole as Achilles’ heel of the biotrophic grapevine pathogen *Plasmopara viticola*. *Protoplasma* 254 1887–1901. 10.1007/s00709-017-1123-y 28550468

[B88] Turchini A. Ferrario L. Popolo L. (2000). Increase of external osmolarity reduces morphogenetic defects and accumulation of chitin in a gas1 mutant of *Saccharomyces cerevisiae*. *J. Bacteriol.* 182 1167–1171. 10.1128/jb.182.4.1167-1171.2000 10648547 PMC94397

[B89] Valery C. Deville-Foillard S. Lefebvre C. Taberner C. Legrand P. Meneau F. (2015). Atomic view of the histidine environment stabilizing higher-pH conformations of pH-dependent proteins. *Nat. Commun.* 6:7771. 10.1038/ncomms8771 PMC4518280 26190377

[B90] Vandelle E. Delledonne M. (2011). Peroxynitrite formation and function in plants. *Plant Sci.* 181 534–539. 10.1016/j.plantsci.2011.05.002 21893249

[B91] Waters E. J. Hayasaka Y. Tattersall D. B. Adams K. S. Williams P. J. (1998). Sequence analysis of grape (*Vitis vinifera*) berry chitinases that cause haze formation in wines. *J. Agric. Food Chem.* 46 4950–4957. 10.1021/jf980421o

[B92] Werner S. Steiner U. Becher R. Kortekamp A. Zyprian E. Deising H. B. (2002). Chitin synthesis during *in planta* growth and asexual propagation of the cellulosic oomycete and obligate biotrophic grapevine pathogen *Plasmopara viticola*. *FEMS Microbiol. Lett.* 208 168–173. 10.1111/j.1574-6968.2002.tb11077.x 11959432

[B93] Whittaker M. Bergmann D. Arciero D. Hooper A. B. (2000). Electron transfer during the oxidation of ammonia by the chemolithotrophic bacterium *Nitrosomonas europaea*. *Biochem. Biophys. Acta* 1459 346–355. 10.1016/s0005-2728(00)00171-7 11004450

[B94] Williams A. C. Edwards H. G. M. (1994). Fourier transform Raman spectroscopy of bacterial cell walls. *J. Raman Spectrosc.* 25 673–677. 10.1002/jrs.1250250730

[B95] Wink D. A. Kasprzak K. S. Maragos C. M. Elespuru R. K. Misra M. Dunams T. M. (1991). DNA deaminating ability and genotoxicity of nitric oxide and its progenitors. *Science* 254 1001–1003. 10.1126/science.1948068 1948068

