# Supplementary material for: Activity and Mechanism of Action of the Bioceramic Silicon Nitride as an Environmentally Friendly Alternative for the Control of the Grapevine Downy Mildew Pathogen Plasmopara viticola
Source: Front Microbiol. 2020 Dec 14;11:610211. doi: 10.3389/fmicb.2020.610211 (PMC7767917; doi:10.3389/fmicb.2020.610211)
Supplement: Supplementary file 1 [file Table_1.docx]

Table S1

| **Band** | **cm^-1^** | **Physical origin** | **Ref.** |
| --- | --- | --- | --- |
| 1 | 482 | Glucose ring vibrations  (Cellulose, glucans) | [S1] |
| 2 | 490 | C-C backbone stretching in polysaccharides | [S1] |
| 3 | 500 | D(+)-mannose | [S2] |
|  |  | Glycine | [S1] |
| 4 | 510 | Cellulose | [S1] |
| 5 | 535 | Ring deformation in trehalose | [S1] |
|  |  | N1-C6-C5 and C2-N3-C4 in-plane ring deformation in adenine | [S3] |
|  |  | D-arabitol | [S4] |
| 6 | 544 | D(+)-trehalose (exocyclic deformation) | [S1] |
|  |  | N3=C4-N4 and C-C=C bending in cytosine | [S5] |
|  |  | D-(-)-ribose | [S6] |
|  |  | Glycerol | [S7] |
| 7 | 558 | β-D-glucose in cellulose | [S1] |
|  |  | In-phase N3-C2=O and N1C2=O bending in cytosine | [S5] |
|  |  | Cholesterol | [S7] |
| 8 | 570 | 6-ring deformation in guanine | [S8] |
|  |  | β-D-glucose in cellulose | [S1] |
| 9 | 583 | C-C-O bending + C-O torsion in cellulose | [S1] |
| 10 | 594 | C2=O bending in cytosine | [S9] |
|  |  | Trilinolenin | [S1] |
|  |  | Glycerol | [S7] |
| 11 | 603 | Trehalose | [S1] |
|  |  | N3-C2=O and N1-C2=O in-phase bending in cytosine | [S5] |
| 11* | 613 | Histidine | [S10] |
| 12 | 623 | C4-C5-N7 – C4-N9-C8 in-plane ring deformation of adenine | [S3] |
|  |  | D-arabitol | [S1] |
|  |  | N-C-C bending in thymine | [S11] |
| 13 | 632 | Out-of-plane C-O-H bend glycerol | [S7] |
| 14 | 643 | Purine ring breathing mode in guanine | [S8] |
|  |  | β-D-glucose in cellulose | [S1] |
|  |  | D-arabitol | [S4] |
| 15 | 649 | β-D-glucose in cellulose | [S1] |
| 15* | 654 | Histidine | [S10] |
| 16 | 669 | C-S stretching | [S12] |
|  |  | Glycerol | [S7] |
| 17 | 681 | Ring breathing in DNA guanine | [S8] |
|  |  | O=CN + CCO bending in ceramides | [S13] |
| 18 | 692 | β-(1,3)-glucan | [S14] |
|  |  | Trehalose | [S1] |
| 19 | 710 | =C-H bending in cellulose | [S1][S15] |
|  |  | Trilinolenin | [S1] |
|  |  | Ring breathing in DNA cytosine | [S5] |
|  |  | D-arabitol | [S4] |
| 20 | 715 | D-arabitol | [S4] |
|  |  | Lecithin | [S1] |
|  |  | C-N stretching in lecithin | [S1] |
| 21 | 731 | Imidazole ring breathing in DNA adenine | [S3] |
|  |  | Trehalose | [S1] |
|  |  | Phosphatidylserine | [S7] |
| 22 | 746 | Ring breathing in DNA thymine | [S11] |
| 23 | 753 | C5-CH_3_ stretching in thymine | [S11] |
| 24 | 764 | Deoxythymidine triphosphate | [S16] |
|  |  | Trehalose | [S1] |
|  |  | O-P-O symmetric stretching in lecithin | [S1] |
| 25 | 782 | *C’*5-O-P-O-*C’*3 phosphodiester symmetric stretching in DNA | [S17] |
| 26 | 795 | Ring breathing in cytosine | [S5] |
| 27 | 807 | 2-deoxy-D-ribose (glucan) | [S6] |
|  |  | Glycerol | [S7] |
|  |  | In-plane ring breathing in uracil | [S9] |
| 28 | 816 | Trioleate | [S1] |
| 29 | 827 | Lecithin | [S1] |
|  |  | O-P-O antisymmetric stretching in lecithin | [S1] |
|  |  | *C’*5-O-P-O-*C’*3 phosphodiester antisymmetric stretching in DNA | [S17] |
| 30 | 837 | Trilinoleate | [S1] |
|  |  | D-dextrose | [S1] |
|  |  | C1-H bending in trehalose | [S1] |
|  |  | β-D-glucose | [S1] |
|  |  | D-arabitol | [S6] |
| 31 | 846 | C4-N9-C8 + N1-C2-N3 and N2-C2-N3 in plane deformation in guanine ring | [S18] |
|  |  | L-(+)-arabinose (glucan) | [S6] |
|  |  | D-(+)-glucose | [S6] |
|  |  | Glycerol | [S7] |
|  |  | C-O, C-C, and C-H bending in trehalose | [S1] |
| 32 | 861 | C-O vibrations in alpha-linolenic acid | [S19] |
| 32* | 872 | Histidine | [S10] |
| 33 | 893 | C-H ring stretching in cellulose | [S1][S15] |
|  |  | Lecithin | [S1] |
|  |  | Trioleate | [S1] |
|  |  | Equatorial C-H bending in β-(1,3)-glucans | [S20] |
|  |  | D-arabitol | [S4] |
| 34 | 906 | D-dextrose | [S1] |
|  |  | Trehalose | [S1] |
|  |  | β-D-glucose | [S1] |
|  |  | D-arabitol | [S4] |
| 35 | 931 | Histidine | [S10] |
|  |  | β-D-glucose | [S1] |
|  |  | D-arabitol | [S4] |
|  |  | C-H bending in arachidonic acid | [S19] |
| 36 | 942 | In-plane ring deformation, N-H vibrations in adenine | [S3] |
|  |  | Trilinolenin | [S1] |
|  |  | D-arabitol | [S4] |
|  |  | Trilinolein | [S1] |
| 37 | 955 | Deoxyadenosine triphosphate | [S16] |
|  |  | Lecithin | [S1] |
|  |  | D-arabitol | [S4] |
|  |  | Glycerol | [S7] |

**References**

[S1] De Gussem K, Vandenabeele P, Verbeken A, Moens L. Raman spectroscopic study of Lactarius spores (Russulales, Fungi). *Spectrochim. Acta Part A* **61:** 2896-2908 (2005).

[S2] She CY, Dinh ND, and Tu AT. Laser Raman scattering of glucosamine, *N*-acetylglucosamine, and glucuronic acid. *Biochim. Biophys. Acta* **372:** 345-357 (1974).

[S3] Lopes RP, Valero R, Tomkinson J, Marques MPM, and Batista de Carvalho LAE. Applying vibrational spectroscopy to the study of nucleobases – adenine as a case study. *New J. Chem.* **37:** 2691-2699 (2013).

[S4] Hedoux A., Guinet Y., Carpentier L., Paccou L., Derollez P., Brandan S.A. Structural and vibrational characterization of sugar Arabinitol structures employing micro-Raman spectra and DFT calculations. *J. Mol. Struct.* **1138**: 118-128 (2017).

[S5] Mathlouthi M, Seuvre AM. F.T.-I.R. and laser-Raman spectra of cytosine and cytidine. *Carbohydr. Res.* **146:** 1-13 (1986).

[S6] Williams A.C., Edwards H.G.M. Fourier transform Raman spectroscopy of bacterial cell walls. *J. Raman Spectrosc.* **25:**673-677 (1994).

[S7] Krafft C, Neudert L, Simat T, Salzer R. Near infrared Raman spectra of human brain lipids. *Spectrochim. Acta* *Part A: Mol. Spectrosc.* **61:** 1529-1535 (2005).

[S8] Mathlouthi M, Seuvre AM. F.T.-I.R. and laser-Raman spectra of guanine and guanosine. *Carbohydr. Res.* **146:** 15-27 (1986).

[S9] Madzharova F, Heiner Z, Guehlke M, and Kneipp J. Surface-enhanced hyper-Raman spectra of adenine, guanine, cytosine, thymine, and uracil. *J. Phys. Chem. C* **120:** 15415-15423 (2016).

[S10] Sonois V, Esteve A, Zwick A, Faller P, Bacsa W. Raman study and DFT calculations of amino acids. *Technical Proceedings* *of the 2008 NSTI Nanotechnology Conference and Trade Show* (NSTI-Nanotech, Nanotechnology 2008) Vol. 1; pp. 352-355.

[S11] Mathlouthi M, Seuvre AM. F.T.-I.R. and laser-Raman spectra of thymine and thymidine. *Carbohydr. Res.* **134:** 23-38 (1984).

[S12] Van Wart H.E., Lewis A., Scheraga H.A., and Saeva F.D. Disulfide bond dihedral angles from Raman spectroscopy. *PNAS* **70(9):** 2619-2623 (1973).

[S13] Mishra S and Tandon P. DFT study of structure and vibrational spectra of ceramide 3: comparison to experimental data. *Mol*. *Simul.* **38(11):** 872-881 (2012).

[S14] Walsh G.M., Leane D., Moran N., Keyes T.E., Forster R.J., Kenny D., and O’Neill S. S-nitrosylation of platelet α_IIb_*β*_3_ as revealed by Raman spectroscopy. *Biochem.* **46:** 6429-6436 (2007).

[S15] Machovic V, Lapcak L, Havelcova M, Borecka L, Novotna Mi, Novotna Ma, Javurkova I, Langrova I, Hajkova S, Brozova A, Titera D. Analysis of European honeybee (*Apis mellifera*) wings using ATR-FTIR and Raman spectroscopy: a pilot study. *Sci* *Agricol. Bohem.* **48(1):** 22-29 (2017).

[S16] D’Amico F., Cammisuli F., Addobbati R., Rizzardi C., Gessini A., Masciovecchio C., Rossi B., and Pascolo L. Oxidative damage in DNA bases revealed by UV resonant Raman spectroscopy. *Analyst* **140:** 1477-1485 (2015).

[S17] Notingher I, Bisson I, Polak JM, Hench LL. In situ spectroscopic study of nucleic acids in differentiating embryonic stem cells. *Vibr. Spectrosc.* **35:** 199-203 (2004).

[S18] Lopes RP, Marques MPM, Valero R, Tomkinson J, Batista de Carvalho LAE. Guanine: a combined study using vibrational spectroscopy and theoretical methods. *Spectrosc. Int. J.* **27(5-6):** 273-292 (2012).

[S19] Czamara K, Majzner K, Pacia MZ, Kochan K, Kaczor A, Baranska M. Raman Spectroscopy of Lipids: A Review. *J. Raman* *Spectrosc.* **46(1):** 4-20 (2015).

[S20] Cael JJ, Koenig JL, and Blackwell J. Infrared and Raman spectroscopy of carbohydrates: Part IV. Identification of configuration- and conformation-sensitive modes for D-glucose by normal coordinate analysis. *Carbohydr. Res.* **32:** 79-91 (1974).
